# MT-ConBiFormer-GPT: multi-target molecular generation for low-data drug discovery via a contrastive BiFormer-GPT architecture and curriculum learning with cross-domain generalization

**DOI:** 10.1093/bib/bbag079

**Published:** 2026-05-11

**Authors:** Romina Norouzi, Karim Abbasi, Parvin Razzaghi, Sajjad Gharaghani

**Affiliations:** Department of Bioinformatics, Kish International Campus, University of Tehran, 16th Azar St, Kish, 1417935840, Iran; Mosaheb Institute for Mathematical Research, Kharazmi University, South Mofateh St, Tehran, 1571914911, Iran; Department of Computer Science and Information Technology, Institute for Advanced Studies in Basic Sciences (IASBS), Prof Yousef Sobouti Blvd, Zanjan, 4513766731, Iran; Laboratory of Bioinformatics and Drug Design (LBD), Institute of Biochemistry and Biophysics, University of Tehran, Enghelab Square, Tehran, 131451365, Iran

**Keywords:** multi-target compounds, generative model, curriculum learning, molecular generation, low-data drug discovery

## Abstract

Multi-target compounds, or polypharmacological agents, hold significant potential for complex diseases like cancer, where single-target therapies are often insufficient. A lack of high-quality bioactivity data limits progress in this field, especially for compounds interacting with multiple proteins simultaneously. This study introduces MT-ConBiFormer-GPT, a deep generative model designed explicitly for low-data, multi-target molecular generation, focusing on the critical PI3K–AKT–mTOR cancer signaling pathway. The framework integrates a variational autoencoder with a BiFormer encoder to capture long-range dependencies in SMILES strings, reducing the quadratic computational complexity associated with standard transformers and mitigating semantic discontinuities. It employs a SMILES-GPT decoder for progressive molecule generation and follows a three-phase training pipeline: unsupervised pre-training, supervised contrastive learning, and curriculum-based fine-tuning. The framework’s efficacy was evaluated through a rigorous, multi-stage assessment. First, the framework was evaluated through benchmarking against state-of-the-art models, with a specialized head-to-head variant, MT-ConBiFormer-GPT_H2H, demonstrating superior performance, thereby validating its generalizability from oncology to neuropsychiatry. An internal ablation study further revealed that the full MT-ConBiFormer-GPT significantly outperformed its baseline, MT-BiFormer-GPT, in both dual- and triplet-target generation tasks, highlighting the advantages of the contrastive learning stage. Additionally, the foundational Base-BiFormer-GPT architecture, a model lacking both the contrastive and curriculum learning stages, highlighted its intrinsic robustness by achieving competitive outcomes in a distinct omics-driven design task. Docking simulations and mechanistic analyses show that the generated molecules, including high-fidelity and scaffold-hopping candidates, display more favorable binding modes than reference inhibitors. This study presents a flexible and computationally efficient framework for multi-target drug discovery in data-limited settings.

## Introduction

The ‘one drug, one target’ approach has been a key strategy in drug discovery [1, 2], but its effectiveness is limited in complex multifactorial diseases like cancer, which disrupt multiple signaling pathways [3, 4]. The PI3K–AKT–mTOR pathway, often dysregulated in human cancers, modulates critical cellular processes such as growth, proliferation, and survival [5]. Mutations in PIK3CA, AKT1, and MTOR drive these aberrations, which are frequently observed in various malignancies [6]. Single-target therapies often fail to deliver clinical success, highlighting the need for strategies that target multiple pathway nodes [7, 8]. Dual inhibition of PIK3CA and AKT1, along with triplet blockade of PIK3CA, AKT1, and MTOR, offers a more comprehensive pathway blockade, potentially overcoming resistance. Precise polypharmacology, which involves designing molecules to modulate multiple protein targets, is a promising but challenging approach to improving therapeutic efficacy and reducing drug resistance [9, 10].

Identifying multi-target candidates within the vast chemical space, estimated at 10 ^23^ to 10 ^60^ drug-like molecules, poses a significant challenge. Deep generative models have become powerful tools for de novo molecular design, enabling exploration well beyond traditional enumeration methods [11, 12]. These models primarily utilize the Simplified Molecular Input Line Entry System (SMILES) representation [13], which frames molecular generation as a language modeling task [14]. SMILES-based deep generative approaches, such as recurrent neural networks (RNNs) [15], variational autoencoders (VAEs) [16], generative adversarial networks (GANs) [17], and Transformer-based models, particularly GPT-like architectures, are pre-trained on large chemical datasets to learn structural syntax and semantics, thereby facilitating the generation of novel molecules with enhanced properties [18].

Generative methods show promise for designing molecules with optimized properties, but most focus on single-target drug discovery. A major challenge in multitarget drug design is the scarcity of high-quality bioactivity data for dual- or multi-target compounds, a stark contrast to the wealth of data available for single-target tasks [19, 20]. This data bottleneck necessitates the development of generative frameworks capable of learning and generalizing efficiently under such data-limited conditions.

Several multitarget generative models have been developed, including DLGN [21] (which integrates adversarial training with reinforcement learning), MTMol-GPT [22] (a Transformer-based model employing generative adversarial imitation learning), RationaleRL [23] (generating molecules from interpretable substructures via reinforcement learning), and CMolRNN [24] (a conditional graph generative model for multi-objective design). However, these methods face significant challenges. For example, DLGN and MTMol-GPT rely on complex training techniques like reinforcement or imitation learning, which are difficult to tune and require large amounts of data. CMolRNN depends on the accuracy of external property predictors to label training data, limiting the generator’s quality based on the predictor’s performance. RationaleRL combines pre-existing chemical substructures, potentially restricting the novelty of generated scaffolds. Recent advancements, such as Alxfuse (which incorporates molecular docking within a reinforcement learning framework for pharmacophore fusion) [25] and DualDiff (a 3D structure-based approach utilizing diffusion models) [26], explore diverse modalities but differ from the generative paradigms of end-to-end SMILES-based chemical language modeling.

Consequently, new strategies are needed to address polypharmacology across diverse chemical spaces under data scarcity. Most current approaches remain limited to generating compounds for two proteins and fail to overcome the fundamental challenge of de novo multi-target molecule generation due to insufficient high-quality multi-target training data [27]. While techniques like transfer learning, temperature sampling, and data augmentation aid low-resource SMILES generation [28], further advances are essential for multitarget tasks. Notably, contrastive learning enhances molecular representation [29], and curriculum learning improves generative efficiency [30]. Deep learning approaches have primarily concentrated on single-target drug discovery, with minimal attention to data-scarce polypharmacological scenarios. To address the limitations of current methods, we present MT-ConBiFormer-GPT.

Our framework is designed to excel in data-scarce scenarios by employing a data-efficient training strategy that avoids the complexities of reinforcement learning and the constraints of 3D structural data. Furthermore, it is one of the pioneering approaches capable of generating molecules that simultaneously target more than two biological targets, a feat enabled by an encoder architecture optimized for computational efficiency.

The proposed model is based on a VAE framework, enabling the capture of structured latent spaces and efficient exploration of sparse chemical landscapes for polypharmacological applications. Its backbone consists of a BioFormer-based encoder for molecular feature extraction and a SMILES-GPT decoder for sequence generation. Generative performance in molecular design relies on molecular representation, with SMILES often used but limited by semantic discontinuity and spatial constraints [31, 32]. While transformers show promise in molecular design, they face challenges with a high computational cost and reduced accuracy for long sequences. Sparse transformers improve efficiency through sparsity. This study introduces the use of sparse vision transformers (ViTs) in molecular synthesis, employing a BiFormer model [33].

Guided by advances like Structured State Space Sequence (S4) models [34], the architecture combines a BiFormer-based encoder for reducing semantic discontinuity and enhancing feature extraction via sparse attention, paired with a SMILES-GPT decoder that generates valid, unique, and synthesizable molecules, often outperforming S4 in performance. Anchored in a VAE framework and informed by prior multi-target bioactivity studies, this architecture enables efficient polypharmacological design [35]. The VAE framework combines the BiFormer encoder and SMILES-GPT decoder to represent and generate complex molecules. While effective for molecular tasks, the proposed multi-stage training strategy, consisting of pre-training, contrastive learning, and curriculum fine-tuning, significantly enhances multitarget molecule generation in data-limited scenarios.

The robustness and versatility of the proposed framework are validated through a comprehensive evaluation. Its competitive performance and adaptability from oncology to neuropsychiatry are demonstrated through comparisons with state-of-the-art models. An internal ablation study shows the superiority of the complete MT-ConBiFormer-GPT model over the MT-BiFormer-GPT, which lacks the supervised contrastive learning stage. Additionally, the Base-BiFormer-GPT architecture, which excludes contrastive and curriculum learning, demonstrates inherent robustness by achieving state-of-the-art results in an omics-driven design task.

This study presents a novel multi-stage generative workflow designed to address data-scarce scenarios by employing (i) pre-training, (ii) supervised contrastive learning, and (iii) curriculum-driven fine-tuning to generate dual-(PIK3CA and AKT1) and triplet-target (PIK3CA, AKT1, and MTOR) ligands. The framework leverages the computationally efficient BiFormer architecture for molecular modeling, achieving competitive performance while maintaining methodological simplicity by circumventing the complexities associated with reinforcement learning. Validation of the framework is conducted through a comprehensive approach, including (i) a competitive benchmark against four state-of-the-art models, (ii) an internal ablation study to confirm the effectiveness of the contrastive learning stage, and (iii) a cross-task generalization study, with the therapeutic potential of the generated molecules further corroborated through *in silico* docking.

## Proposed method

**Figure 1:**
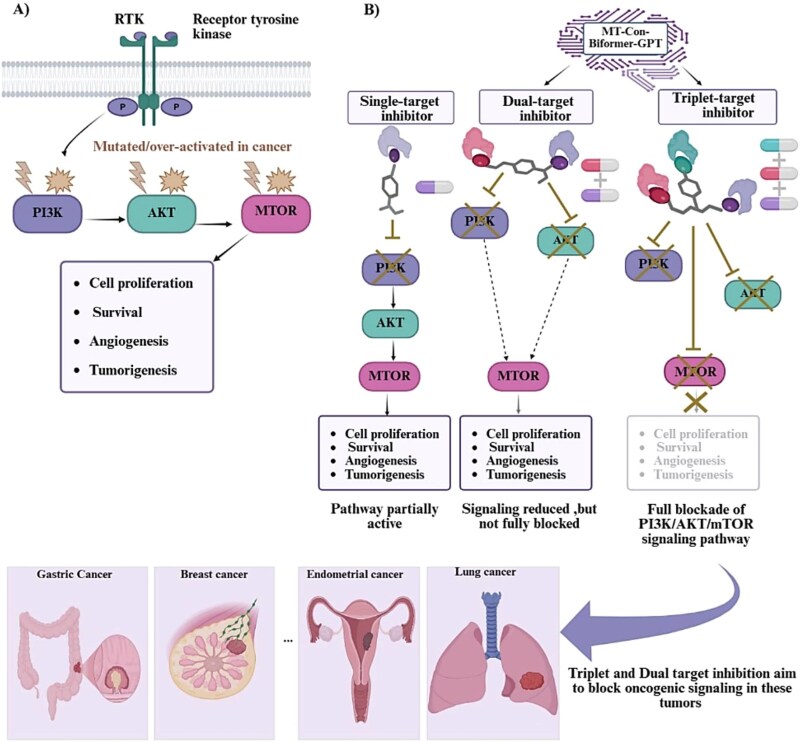
The PI3K–AKT–mTOR signaling pathway and multi-target inhibition strategy. (A) PI3K–AKT–mTOR signaling axis, where oncogenic activation of PI3K, AKT, or MTOR drives cell proliferation, survival, angiogenesis, and tumorigenesis. (B) The conceptual architecture of MT-ConBiFormer-GPT demonstrates a novel approach for generating dual- and triplet-target inhibitors targeting PI3K, AKT, and mTOR; in marked contrast to traditional single-target therapeutic agents that often yield only partial pathway suppression, these multi-target ligands aim to achieve a significantly deeper and more efficacious blockade of the PI3K/AKT/mTOR cascade to combat gastric, breast, endometrial, and lung cancers.

**Figure 2:**
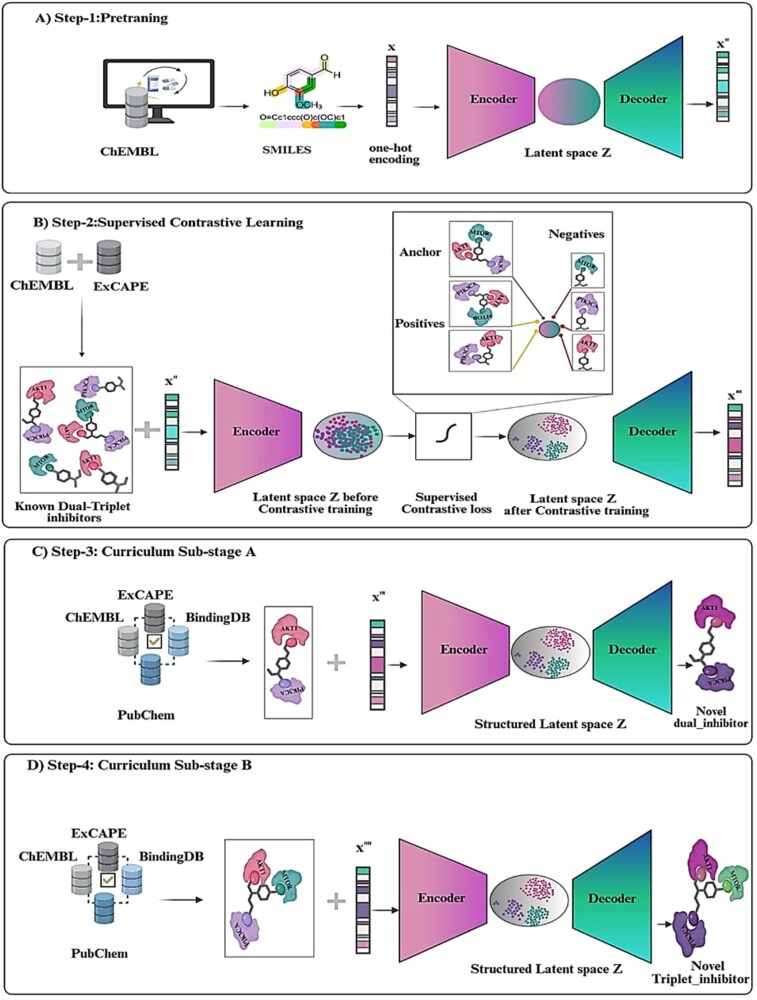
Overview of the MT-ConBiFormer-GPT multi-target molecule design workflow. The workflow consists of (A) unsupervised pre-training, (B) supervised contrastive learning, (C) dual-target fine-tuning, and (D) triplet-target fine-tuning.

We developed MT-ConBiFormer-GPT, a deep generative framework for de novo, multi-target molecular design in low-data scenarios. While validated on the PI3K–AKT–mTOR cancer pathway (Fig. 1), the framework is designed to be target-independent. Our methodology (Fig. 2) follows a four-stage training pipeline. First, we conduct unsupervised pre-training (Fig. 2A) to capture general molecular syntax. Next, we apply supervised contrastive learning (Fig. 2B) to organize the latent space according to pharmacological profiles. Finally, we implement a curriculum-based fine-tuning phase that progressively adapts the model for dual-target generation (Fig. 2C) and, subsequently, triplet-target generation (Fig. 2D).

**Figure 3:**
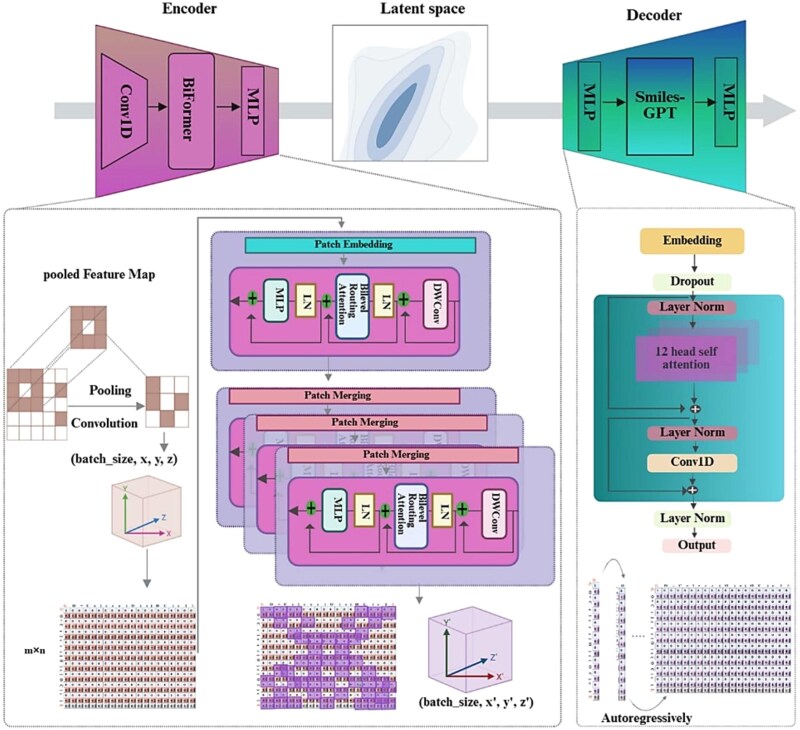
Schematic overview of the MT-ConBiFormer-GPT architecture.

The framework’s architecture (Fig. 3) is a VAE that pairs a novel BiFormer-based encoder with a robust SMILES-GPT decoder. The encoder’s key advantage is its sparse attention mechanism, which efficiently models long-range dependencies in SMILES strings while reducing computational complexity from the quadratic $\mathrm{O} ({\mathrm{N}}^2)$ of standard attention to a more scalable $\mathrm{O}({\mathrm{N}}^{4\beta})$ [33]. The pre-trained decoder SMILES-GPT then autoregressively generates molecules from the structured latent space. The problem formulation, model architecture, multi-stage training protocols, dataset creation, and evaluation metrics are thoroughly described in Supplementary Information (Sections S1–S5).

## Results

The evaluation of the MT-ConBiFormer-GPT framework begins with a comparative analysis against state-of-the-art models. An ablation study follows, contrasting the generative performance of the full framework with its ablated baseline, MT-BiFormer-GPT, for dual- and triplet-target inhibitors. *In silico* validation for the PI3K-AKT–mTOR task includes scaffold analysis and docking. The section concludes by showcasing the adaptability of the core architecture Base-BiFormer-GPT through a cross-task generalization study on an omics-driven drug design benchmark.

### Benchmark against state-of-the-art models

The evaluation of the proposed framework against state-of-the-art methods involved a benchmark on the DRD2/HTR1A dual-target task, prioritized for neuropsychiatric disorders like schizophrenia. It included a general comparison with four leading models: CMolRNN, RationaleRL, DLGN, and MTMol-GPT, and a specialized head-to-head (H2H) benchmark with the MT-ConBiFormer-GPT_H2H variant against MTMol-GPT, identified as the top-performing competitor. The H2H approach was explicitly designed to account for training paradigm differences. Unlike methods reliant on reinforcement or imitation learning, the contrastive learning framework utilized in this study necessitated labeling dual-target data.

**Figure 4:**
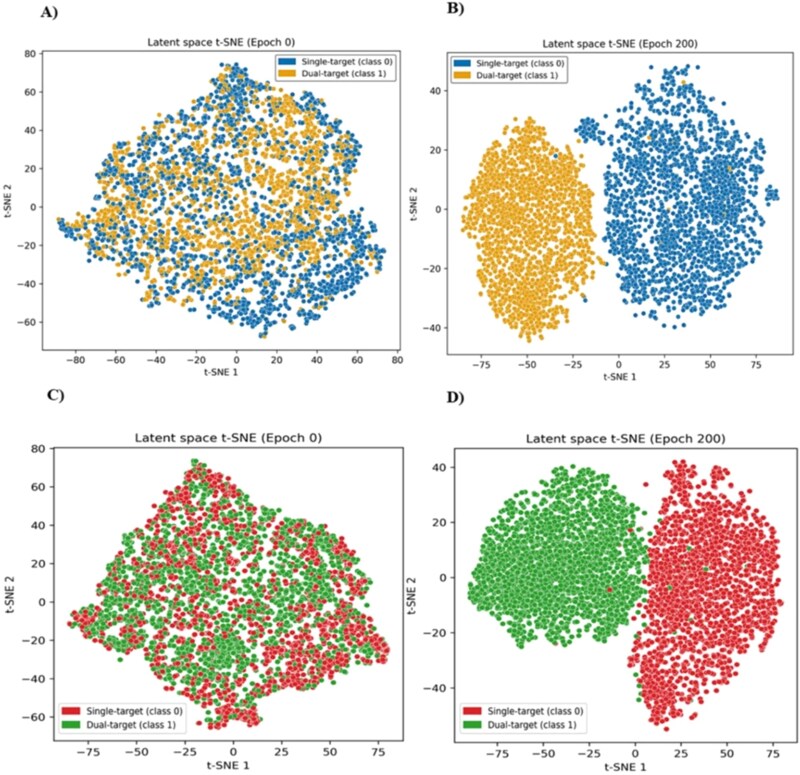
t-SNE visualization of latent space embeddings demonstrating the impact of supervised contrastive learning. Panels (A) and (B) show the general benchmark’s latent space before (Epoch 0) and after (Epoch 200) training, respectively, illustrating the separation of single-target (blue) and dual-target (orange) classes. Panels (C) and (D) display the MT-ConBiFormer-GPT_H2H benchmark’s latent space before (Epoch 0) and after (Epoch 200) training, highlighting the distinct clustering of single-target (red) and dual-target (green) molecules.

Consequently, the H2H benchmark employed MTMol-GPT’s single-target training data as the negative class and a high-confidence set of its dual-target-generated molecules as the positive class, ensuring a rigorous and fair comparison. Before generation, the crucial contrastive learning stage was validated. The model successfully transformed initially overlapping latent spaces into distinct, well-separated clusters for both the general (Fig. 4A and B) and H2H (Fig. 4C and D) setups. As quantified in Table 1 (Supplementary Table S1), this resulted in high classification accuracies of 0.9922 (General) and 0.9952 (H2H). Full experimental details are available in the Supplementary Information (Section S6).

#### Comparative performance on multi-target generation

As a result of validating latent space discriminability, the MT-ConBiFormer-GPT framework’s generative performance on the DRD2/HTR1A dual-target task was evaluated using standardized MOSES metrics, based on a benchmarking protocol for state-of-the-art models. Both the general (MT-ConBiFormer-GPT) and Head-to-Head (MT-ConBiFormer-GPT_H2H) versions demonstrated strong performance as illustrated in Table 2 (Supplementary Table S2), with the MT-ConBiFormer-GPT_H2H demonstrating perfect validity (1.000) and outperforming all other methods, while both the H2H and general versions exhibited perfect novelty (1.000). The framework exhibited superior capability in exploring diverse chemical spaces, achieving the highest Internal Diversity (IntDiv) scores in both the general (0.887) and H2H (0.889) benchmarks.

**Figure 5:**
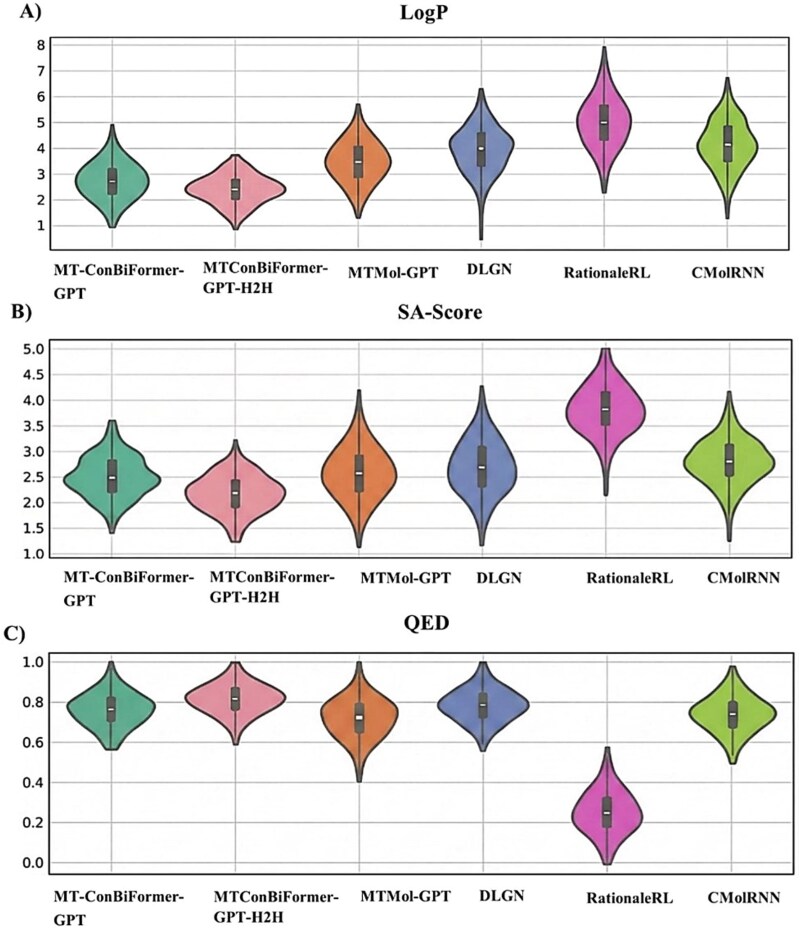
Physicochemical property benchmark. The violin plots visualize the distribution of (A) LogP, (B) SA score, and (C) QED scores. The results confirm that the generated compounds exhibit physicochemical properties consistent with drug-like molecules and competitive with leading generative frameworks.

Our approach is robust at reproducing key structural features of known inhibitors for the DRD2 target, the MT-ConBiFormer-GPT_H2H model achieved the highest scores for Fragment (0.9821) and Scaffold (0.50887) similarity and the best (lowest) Fréchet ChemNet Distance (FCD) of 5.5283. For the HTR1A target, the MT-ConBiFormer-GPT model demonstrated top performance, with the highest scores for SNN (0.4853), Frag (0.9891), and Scaff (0.4165) similarity. The quality of the generated molecules was assessed by analyzing the distributions of key physicochemical properties: QED, LogP, and SA. Molecules generated by the framework under both benchmark setups exhibited drug-like properties competitive with state-of-the-art models, as shown in Fig. 5, with QED scores near 0.8, LogP distributions within 2.0–5.0, and low synthetic accessibility (SA) scores below 3.0, suggesting easy synthesis. The MT-ConBiFormer-GPT_H2H variant’s performance, validated in a direct H2H comparison with MTMol-GPT, highlights its competitive edge in multi-target molecular design, producing candidates that meet critical drug development criteria.

### Performance of inference stages

This section evaluates the MT-ConBiFormer-GPT framework within the PI3K–AKT–mTOR pathway, starting with an analysis of the initial training phases that establish the model’s foundational capabilities. A rigorous ablation study compares the generative performance of the full framework with MT-BiFormer-GPT. The multi-stage training strategy is essential for producing novel, diverse, and structurally relevant molecules, as shown by the comparative analysis for dual- and triplet-target generation.

#### Reconstruction accuracy and latent consistency in unsupervised pre-training

Each SMILES string was represented as a one-hot encoded tensor of shape (100, 48) using a 48-character vocabulary, with padding or truncation to 100 characters for a standardized and lossless format. The pre-training phase on a diverse molecular dataset showed stable convergence and robust learning dynamics (Supplementary Fig. S12), establishing MT-ConBiFormer-GPT’s ability to learn chemically meaningful latent representations and accurately reconstruct valid SMILES. This forms the foundation for subsequent contrastive learning and curriculum-based multitarget fine-tuning.

#### Latent discriminability in supervised contrastive learning

Supervised contrastive learning was applied after pre-training to refine the latent space, enhancing its ability to distinguish single-target from multitarget molecular profiles, particularly for PI3K–AKT–mTOR pathway inhibitors. A hybrid loss combining contrastive and reconstruction objectives was optimized over 200 epochs, achieving stable convergence (Supplementary Fig. S13). A logistic regression classifier applied to the frozen latent vectors attained 99.99% classification accuracy in Table 3 (Supplementary Table S3) with nearly perfect precision, recall, and F1 scores.

**Figure 6:**
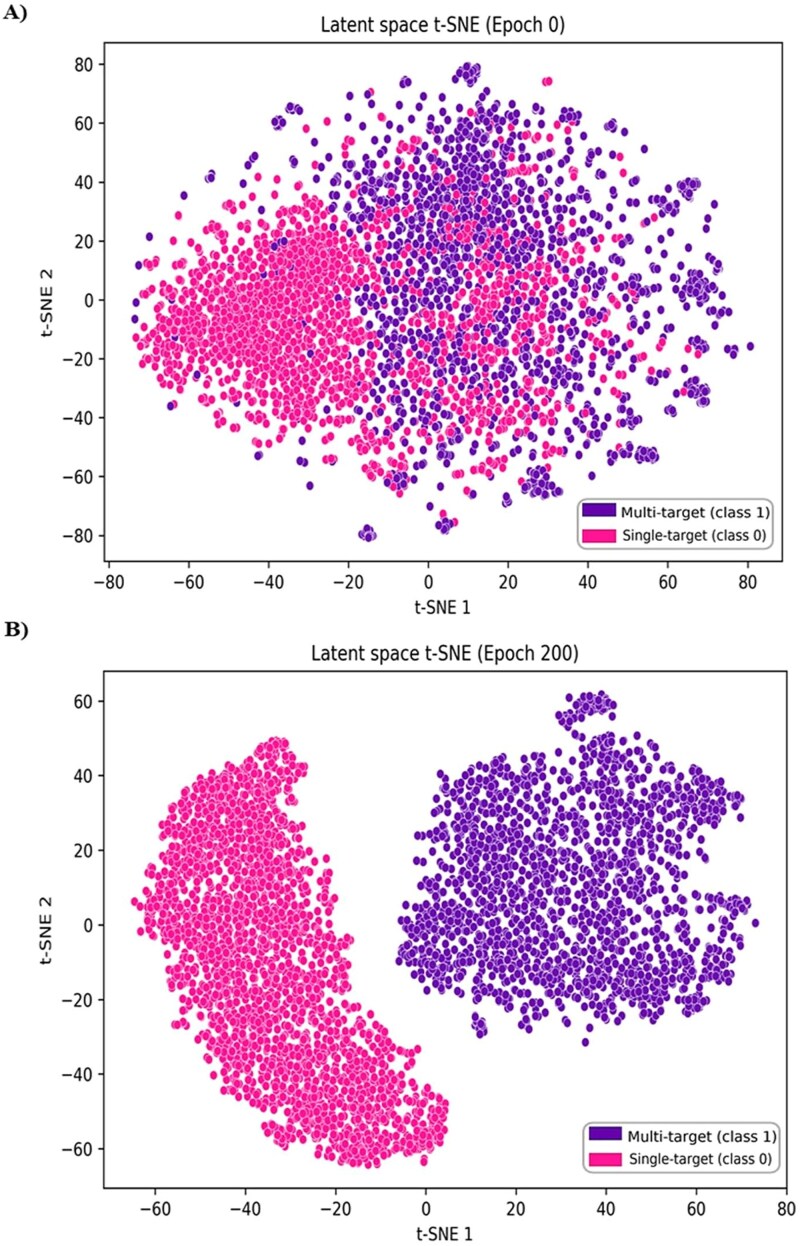
Evolution of the latent space for single- versus multi-target (dual/triplet) inhibitors. The t-SNE projections show (A) significant class overlap at Epoch 1 and (B) the formation of distinct, well-separated clusters by Epoch 200. The emergence of the distinct multi-target cluster (pink) confirms the model’s ability to distinguish potential dual- and triplet-target pharmacological profiles from single-target compounds (purple).

The confusion matrix (Supplementary Fig. S14) confirmed the model’s exceptional accuracy in distinguishing single- and multitarget molecules, with strong alignment between predicted and actual labels. A comparative t-SNE visualization of the latent space (Fig. 6A and B) showed a transition from overlapping to well-separated clusters after contrastive learning, corresponding to the two molecular target profiles. These results demonstrate that contrastive learning effectively organizes the latent space to capture meaningful polypharmacological differences, providing a robust foundation for curriculum-guided fine-tuning.

#### Ablation study of generative performance after curriculum-guided fine-tuning

This section presents findings from a structured curriculum-guided fine-tuning approach targeting dual and triplet objectives within the PI3K–AKT–mTOR pathway. The fine-tuning process was conducted under minimal data conditions, using only 5 unique dual-target and 16 unique triplet-target seed molecules (Supplementary Figs S5 and S6). The generative performance of MT-ConBiFormer-GPT, a contrastively enhanced model, is compared with its ablated baseline counterpart, MT-BiFormer-GPT, which lacks contrastive shaping, for dual- and triplet-target generation tasks. Key performance metrics from the MOSES benchmark, including validity, uniqueness, novelty, and structural similarity, highlight the incremental benefits of supervised contrastive learning in structuring the latent chemical space and enhancing multi-target molecular generation.

▪ Curriculum sub-stage stage I: dual-target fine-tuning (PIK3CA + AKT1)

In the dual-target task, the MT-ConBiFormer-GPT variant significantly outperformed the ablated baseline across all key metrics, as detailed in Table 4 (Supplementary Table S4). MT-ConBiFormer-GPT model achieved higher uniqueness while maintaining excellent validity and novelty. Its structural similarity to known active compounds improved significantly, as shown by a lower FCD and a higher Similarity to Nearest Neighbor (SNN). This indicates that the contrastively structured latent space effectively directs the generator toward pharmacologically relevant chemical regions.

▪ Curriculum Sub-stage II: Triplet-Target Fine-Tuning (PIK3CA + AKT1 + MTOR):

The transition to the more challenging triple-target task underscores the enduring effectiveness of the contrastive learning stage. The MT-ConBiFormer-GPT model consistently outperformed the ablated baseline in uniqueness and internal diversity while achieving a lower FCD score, reflecting an optimal balance between distributional fidelity (low FCD) and exploratory diversity (high IntDiv). These results validate the generalizability of the contrastive curriculum learning strategy in dual- to triple-target regimes, enhancing the model’s capacity to generate high-quality candidates without compromising diversity.

#### Physicochemical property profiling of generated ligands

**Figure 7:**
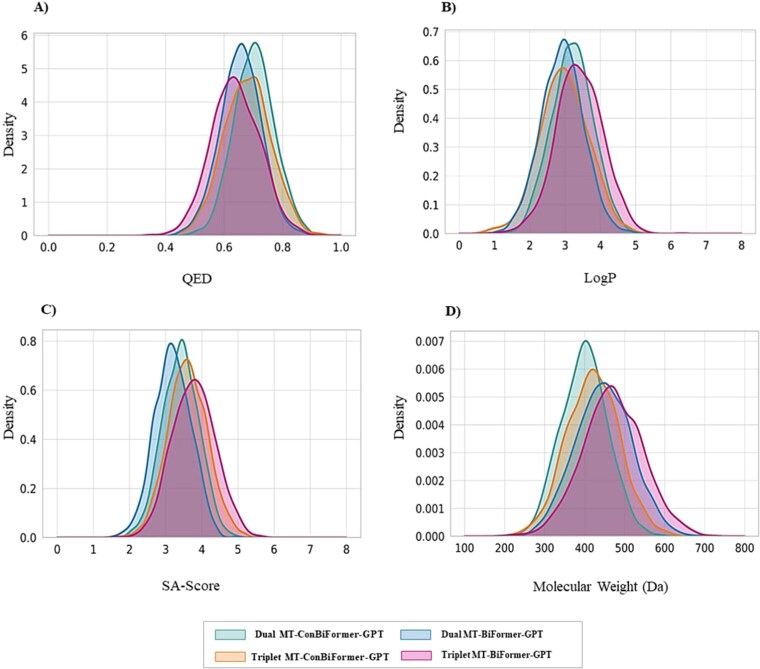
Physicochemical property distributions of two model variants for dual- and triplet-target tasks. These density plots compare the distributions of (A) QED, (B) LogP, (C) SA score, and (E) MW for the full MT-ConBiFormer-GPT model against its ablated baseline on the PI3K–AKT–mTOR pathway.

The physicochemical properties (QED, LogP, SA score, and MW) of the generated molecules were assessed to evaluate their medicinal chemistry viability, as shown in Figure 7.

The performance of the full MT-ConBiFormer-GPT model was compared with its ablated baseline across dual- and triplet-target tasks for the PI3K–AKT–mTOR pathway, revealing that all model variants produce molecules with drug-like properties suitable for further development. Analysis of the property distributions indicates that all model variants generate molecules with robust drug-like profiles. QED scores are concentrated in the favorable 0.6–0.75 range, and LogP values are tightly centered around 3.0, highlighting high drug-likeness and bioavailability across conditions. A clear progression is observed from dual- to triplet-target tasks, with distributions for Molecular Weight (MW) and SA score shifting slightly higher, reflecting the increased structural complexity required to target a third molecule.

Notably, the full MT-ConBiFormer-GPT model demonstrates a consistent, subtle improvement, generating molecules with marginally lower (improved) SA scores compared to its ablated baseline in both dual and triplet settings. This suggests that the contrastive learning stage not only enhances generative metrics but also refines the production of more synthetically accessible compounds.

#### Diversity and novelty of scaffolds

A Murcko scaffold analysis was used to quantify how strongly the fine-tuning data constrain the chemical space and how far MT-ConBiFormer-GPT can move beyond these chemotypes. Both the dual- and triplet-target datasets exhibit significant scaffold imbalance; a highly restricted set of Murcko scaffolds constitutes the vast majority of molecules. In each task, a single dominant chemotype prevails, while the remainder comprises only a few closely related variants (Supplementary Figs S7A and S8A). The full MT-ConBiFormer-GPT model demonstrated superior scaffold generation, outperforming its ablated baseline in the dual-target task (4584 versus 4479 unique scaffolds) and, more significantly, the triplet-target task (4584 versus 4243). With an 8% increase in scaffold novelty for the triplet objective, the results highlight the importance of the supervised contrastive learning stage in broadening the model’s ability to explore diverse chemical space.

Supplementary Figs S7B–C and S8B–C further elucidate these effects at the scaffold level. For each task, panel A presents the four most frequent Murcko scaffolds from the fine-tuning sets, while panels B and C compare these references against the best-matching scaffolds generated by both the MT-BiFormer-GPT and MT-ConBiFormer-GPT models. These are annotated with their ECFP4-based Tanimoto similarity. The comparison reveals that while both models successfully regenerate the dominant training chemotypes, MT-ConBiFormer-GPT typically produces variants with slightly lower similarity scores that nonetheless preserve the essential core topology. This distinct pattern indicates that the contrastive learning stage actively promotes scaffold-hopping (SH) analog by generating novel structures that retain the key pharmacophoric framework rather than simply memorizing the exact scaffolds present in the fine-tuning data.

**Figure 8:**
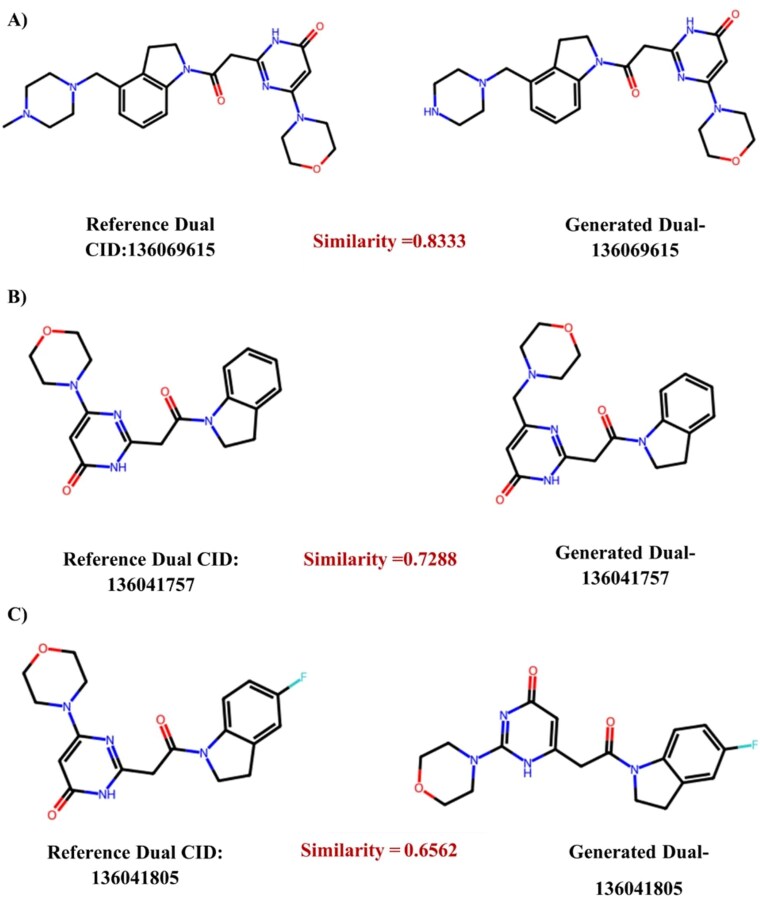
Representative structural recovery for dual-target generation. Comparison of generated molecules with their nearest structural neighbors from the dual-target reference set. The model demonstrates HF recovery of training scaffolds (A, B, similarity >0.70), successfully preserving the fused bicyclic core and linker orientation characteristic of the reference inhibitors, and generates SH analog (C, similarity 0.66) that retain these key spatial features while diversifying the chemical structure.

To complement the scaffold diversity metrics and provide structural interpretation, we conducted a pairwise similarity analysis to identify representative generated structures for the dual-target task. We compared the generated library against the dual-target reference set using fingerprint-based Tanimoto similarity to verify the retention of key pharmacophoric features. As illustrated in Fig. 8, MT-ConBiFormer-GPT successfully reconstructs complex molecular architectures found in known dual inhibitors while introducing rational structural variations. In the high-fidelity (HF) examples (Similarity 0.83), the model accurately reproduces the central fused heterocyclic scaffold and the critical terminal saturated ring with only minor variations in the saturation of the N-linked system. For linker modifications (Similarity 0.73), the generated analogue preserves the essential hydrogen-bond acceptor motifs but introduces a bioisosteric modification to the linker region, slightly altering the spatial orientation of the terminal aromatic ring. Furthermore, the model demonstrates true scaffold hopping (Similarity 0.66) by modifying the fused-ring core; while the reference utilizes a specific halogenated derivative, the generated molecule retains the essential hinge-binding geometry while exploring a non-halogenated, alternative fused-ring architecture.

**Figure 9:**
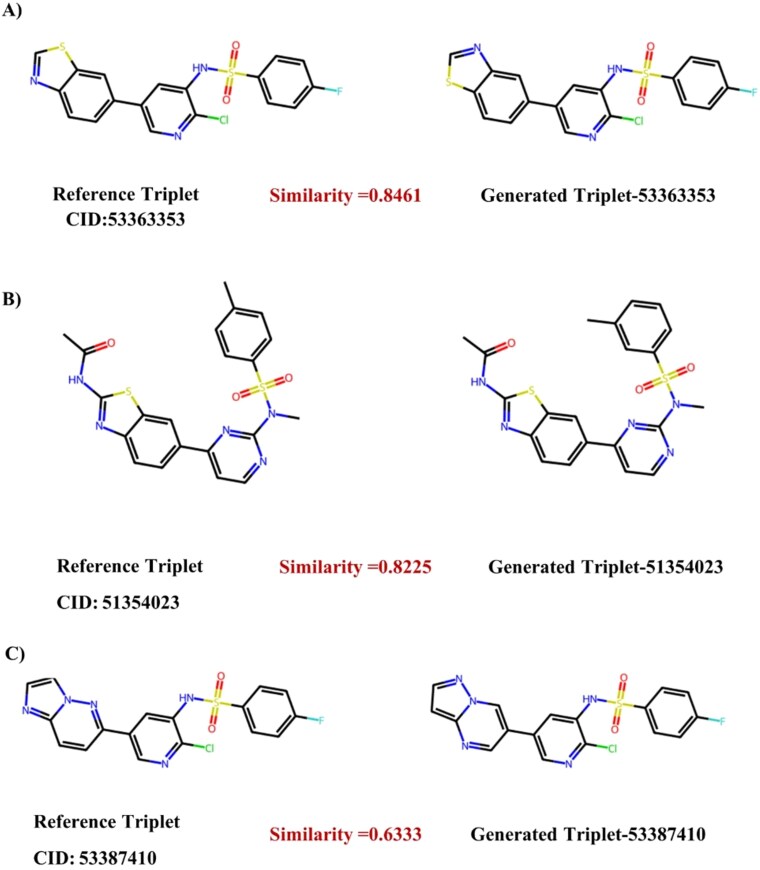
Representative structural recovery for triplet-target generation. Comparison of generated molecules with their nearest structural neighbors from the triplet-target reference set. The model successfully reconstructs complex fused-ring systems and sulfonamide pharmacophores with high fidelity (A and B, similarity >0.80) and demonstrates successful scaffold hopping (C, similarity 0.63), retaining essential binding features while diversifying the core architecture.

A similar structural analysis was conducted for the triplet-target generation task, as shown in Fig. 9, where the model exhibits remarkable fidelity in reconstructing the highly complex, fused-ring systems characteristic of triplet inhibitors. The model demonstrates exact pharmacophore retention (Similarity 0.85) by precisely replicating the bicyclic heteroaromatic core and the sulfonamide linker, which is the critical pharmacophore for this profile, with variations primarily occurring in the substitution pattern on the terminal phenyl ring. Regarding side-chain optimization (Similarity 0.82), the model retains the complex urea/sulfonamide connectivity but modifies the alkyl substitution on the central ring, suggesting an exploration of hydrophobic fit within the binding pocket.

Finally, the analysis highlights core scaffold variation (Similarity 0.63), where the generated analogue replaces the reference system with an alternative fused heterocyclic core while strictly preserving the geometry of the sulfonamide tail required for the tri-kinase interaction. These results confirm that the model captures the underlying pharmacophoric logic of the training data, allowing it to generate valid, high-affinity analogues that are distinct from the training set.

#### *In silico* validation of multi-target binding potential

A molecular docking study was conducted to assess the multi-target binding potential of the generated molecules. Virtual screening and docking protocols were applied to approximately 10,000 unique and valid molecules generated by the top-performing MT-ConBiFormer-GPT model. Crystal structures for the study were sourced from the Protein Data Bank [36], including PIK3CA (PDB: 4JPS), AKT1 (PDB: 4EKL), and mTOR (PDB: 4JSV). Proteins were prepared using AutoDock Tools by removing water molecules, incorporating polar hydrogens, and assigning Kollman charges. Ligands were prepared by generating 3D conformers with all torsional bonds treated as rotatable and assigning Gasteiger charges. Docking simulations were conducted using AutoDock Vina [37], employing a grid box centered on the active site of each protein, with binding affinities determined based on the top-ranked docking scores (kcal/mol).

To avoid arbitrary cherry-picking and to make the docking set representative of the generative model, we applied a four-stage virtual-screening cascade to the generated library. First, molecules failing basic chemical-validity checks or Lipinski’s rule-of-five were removed. Second, we retained only molecules whose Bemis–Murcko scaffolds had ECFP4 Tanimoto similarity ≥0.50 to at least one known inhibitor in the dual- or triplet-target fine-tuning sets, ensuring that candidates contained plausible kinase-inhibitor pharmacophores. Third, the remaining molecules were ranked by a high quantitative estimate of drug-likeness (QED) and low SA score to prioritize medicinally attractive scaffolds. Finally, Butina clustering on ECFP4 fingerprints was used to select diverse representatives from the top-ranked pool.

From these enriched clusters, four representative generated ligands were selected for detailed docking and interaction analysis: two for the dual-target task and two for the triplet-target task. For each task, we selected one HF candidate, which retains the core scaffold of a top-performing reference inhibitor, and one SH candidate, which introduces a modified core while preserving pharmacophoric features.

To rigorously validate their binding potential, each generated candidate was docked alongside its specific corresponding reference inhibitor (Ref-HF and Ref-SH) against the relevant protein targets. For the dual-target task, the generated HF candidate Dual-HF achieved docking scores of −9.7 kcal/mol (PIK3CA) and − 10.1 kcal/mol (AKT1). These values surpass its direct reference inhibitor, Ref-Dual-HF (−9.5 and − 10.0 kcal/mol), as well as the mean affinity of the five dual-target reference molecules (−9.3 and − 9.6 kcal/mol). Similarly, the SH candidate Dual-SH demonstrated strong multi-target affinity with scores of −9.3 kcal/mol (PIK3CA) and − 9.6 kcal/mol (AKT1), outperforming its corresponding reference Ref-Dual-SH (−9.1 kcal/mol for both targets) (Supplementary Table S5). For the more challenging triplet-target task, the generated candidates maintained robust binding across all three kinase targets. The HF candidate Triplet-HF exhibited a well-balanced profile with affinities of −9.4 kcal/mol (PIK3CA), −9.5 kcal/mol (AKT1), and − 10.0 kcal/mol (mTOR). These scores are highly comparable to its reference Ref-Triplet-HF (−9.3, −9.3, and − 10.0 kcal/mol). Likewise, the SH candidate Triplet-SH achieved scores of −9.4 kcal/mol (PIK3CA), −9.5 kcal/mol (AKT1), and − 10.0 kcal/mol (mTOR), effectively matching the performance of Ref-Triplet-SH (Supplementary Table S6).

**Figure 10:**
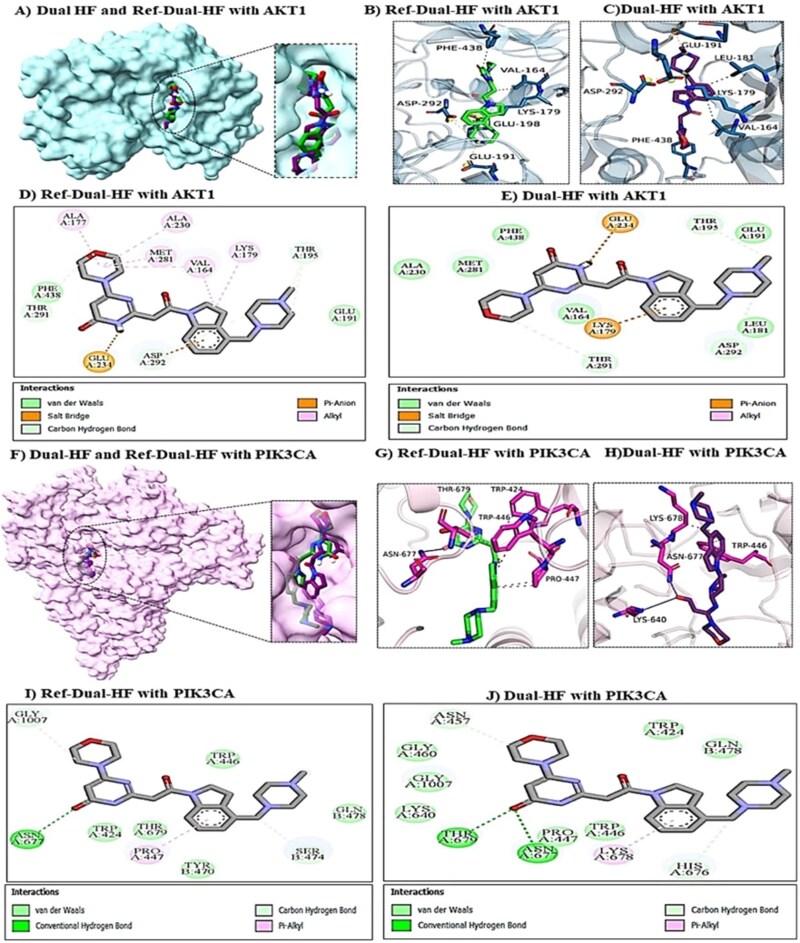
Mechanistic binding mode comparison of the generated candidate dual-HF versus the reference inhibitor ref-dual-HF. (A and F) Structural superpositions in AKT1 and PIK3CA active sites confirming high shape complementarity. (B–E) AKT1 interactions: while the reference inhibitor relies on weak hydrophobic contacts (D), dual-HF anchors firmly via a hydrogen bond with Val164 and establishes a strong electrostatic pi-anion interaction with the catalytic residue Lys179 (E), explaining its superior affinity. (G–J) PIK3CA interactions: dual-HF engages the critical ATP-binding residue Lys640 via a specific hydrogen bond (J), an interaction completely absents in the reference inhibitor (I), confirming the model’s ability to optimize binding geometry for multiple targets.

To elucidate the structural basis of these affinities, we analyzed non-covalent protein-ligand interactions using PLIP [38], BIOVIA Discovery Studio Visualizer [34] was employed to generate detailed 2D interaction maps [39]. A mechanistic analysis of the binding modes confirmed that the top candidates achieve their high binding affinities by successfully recapitulating the binding patterns of the known reference inhibitors. For the dual-target task in AKT1, the superposition of the generated molecule Dual-HF (purple) and the reference Ref-Dual-HF (green) confirms that both ligands occupy the same ATP-binding cleft (Fig. 10A).

However, detailed interaction analysis reveals that Dual-HF outperforms the reference by targeting the critical catalytic residue Lys179. While the reference only forms a non-specific hydrophobic Pi-Alkyl interaction with this residue (Fig. 10D), Dual-HF establishes a strong electrostatic Pi-Anion interaction (Fig. 10E), a hallmark of potent kinase inhibition. This enhanced binding is structurally visible in the 3D interaction diagrams, where Dual-HF is deeply embedded and bridged by key residues Glu191 and Lys179 (Fig. 10C), whereas the reference lacks this specific anchor (Fig. 10B). Furthermore, Dual-HF exhibits superior shape complementarity, evidenced by a dense network of Van der Waals contacts with residues such as Phe438, Ala230, Met281, and Val164 (Fig. 10E), indicating a snug fit that minimizes steric clashes compared to the reference.

In the PIK3CA active site, the structural overlay (Fig. 10F) again validates that both ligands target the same hydrophobic pocket, but Dual-HF adopts a conformation that facilitates distinct high-quality hydrogen bonding. Crucially, Dual-HF forms a specific hydrogen bond with Lys640 (Fig. 10H and J), a key residue often involved in ATP binding, whereas the reference inhibitor completely fails to engage this target (Fig. 10G and I). Additionally, Dual-HF is stabilized by a hydrogen bond network involving Thr679 and Asn677(Fig. 10J), providing a firmer anchor in the pocket center compared to the reference (Fig. 10I). This specific engagement is complemented by a richer set of non-polar contacts (e.g., Asn457, Gly460, Pro447), confirming that the generated molecule achieves its high docking score through a mechanistically superior binding mode characterized by critical catalytic residue targeting and optimized geometric fit (Fig. 10G versus h). The generated SH candidate Dual-SH also demonstrated superior binding characteristics compared to its reference Ref-Dual-SH (Supplementary Fig. S15). In AKT1, Dual-SH established three conventional hydrogen bonds with Thr160, Phe161, and Lys179 and formed strong attractive charge interactions with Glu198 and Asp292 (Supplementary Fig. S15B). In contrast, the reference lacked these hydrogen bonds and exhibited an unfavorable repulsive interaction with Lys179 (Supplementary Fig. S15A).

**Figure 11:**
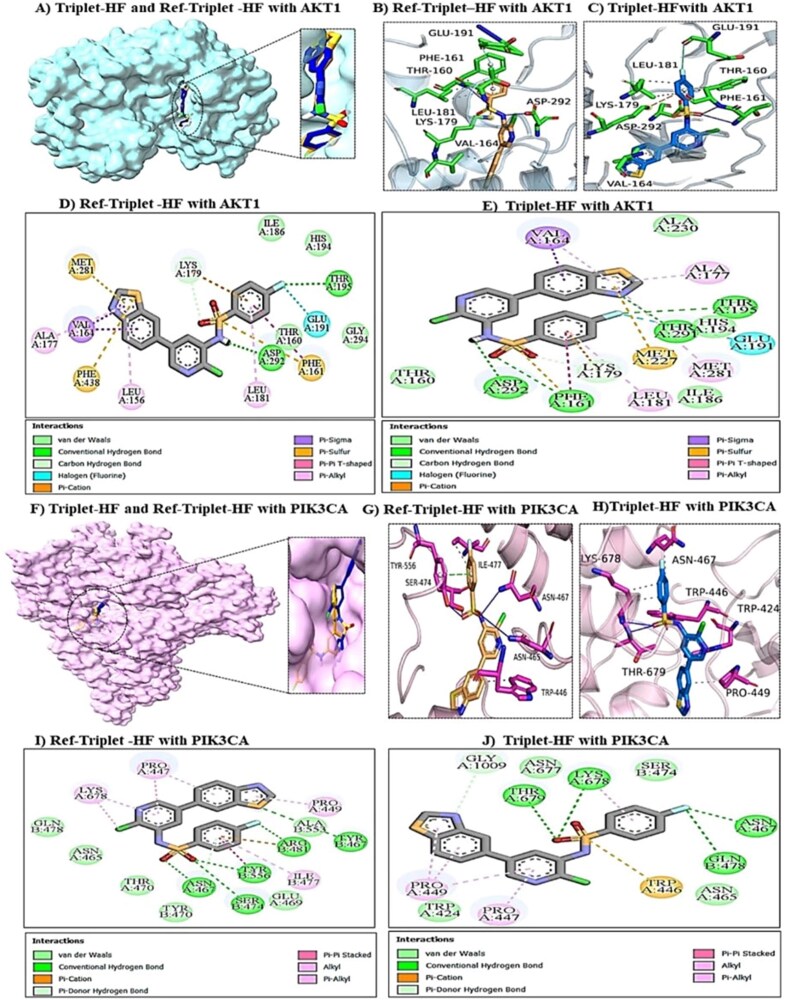
Compares the binding modes of triplet-HF and ref-triplet-HF in AKT1 and PIK3CA. Structural superpositions (A and F) confirm both ligands occupy the same ATP-binding clefts. In AKT1 (B–E), triplet-HF establishes a robust network of conventional hydrogen bonds with Lys179, Asp292, Glu191, and Thr195, providing a superior geometric lock compared to the reference’s reliance on cation interactions. In PIK3CA (g–j), triplet-HF distinguishes itself by forming strong hydrogen bonds with Lys678 and Thr679, anchoring interactions that are notably absent in the reference inhibitor Fig. 12—mechanistic binding analysis of triplet-HF versus ref-triplet-HF in the mTOR active site. (A) Surface representation showing the generated triplet-HF (blue) and reference (orange) occupying the deep ATP-binding pocket of mTOR. (B and C) 3D interaction details. Triplet-HF (C) penetrates deeply into the binding cleft, engaging Thr2279 and Asn46 more effectively than the reference (B). (D and E) 2D interaction maps: a critical distinguishing feature is the formation of specific halogen bonds (cyan lines) between triplet-HF and residues Thr2279 and Asn132 (E). Combined with a denser network of van der Waals contacts (light green circles), this explains the generated candidate’s high predicted binding affinity compared to the reference, which lacks these specific halogen-mediated stabilizing interactions (D).

Similarly, in PIK3CA, Dual-SH secured a strong, attractive charge interaction with Asp933 and a specific Pi-Sulfur contact with Met922 (Fig. S15D), interactions that were absent in the reference (Fig. S15C). These results underscore the model’s ability to generate novel scaffolds that not only retain but also mechanistically improve upon the binding features of known inhibitors. This conservation of binding pattern was also evident in the more challenging triplet-target task. The generated molecule Triplet-HF and the reference inhibitor Ref-Triplet-HF consistently form essential interactions with key hinge or catalytic residues across all three kinases. In the AKT1 active site (Fig. 11A), the superposition confirms that Triplet-HF (blue) and the reference (orange) occupy the same ATP-binding cleft with significant spatial alignment. However, detailed interaction analysis reveals that Triplet-HF anchors itself via a considerably more robust network. It establishes conventional hydrogen bonds with Lys179, Asp292, Glu191, His194, and Thr195 (Fig. 11E).

**Figure 12:**
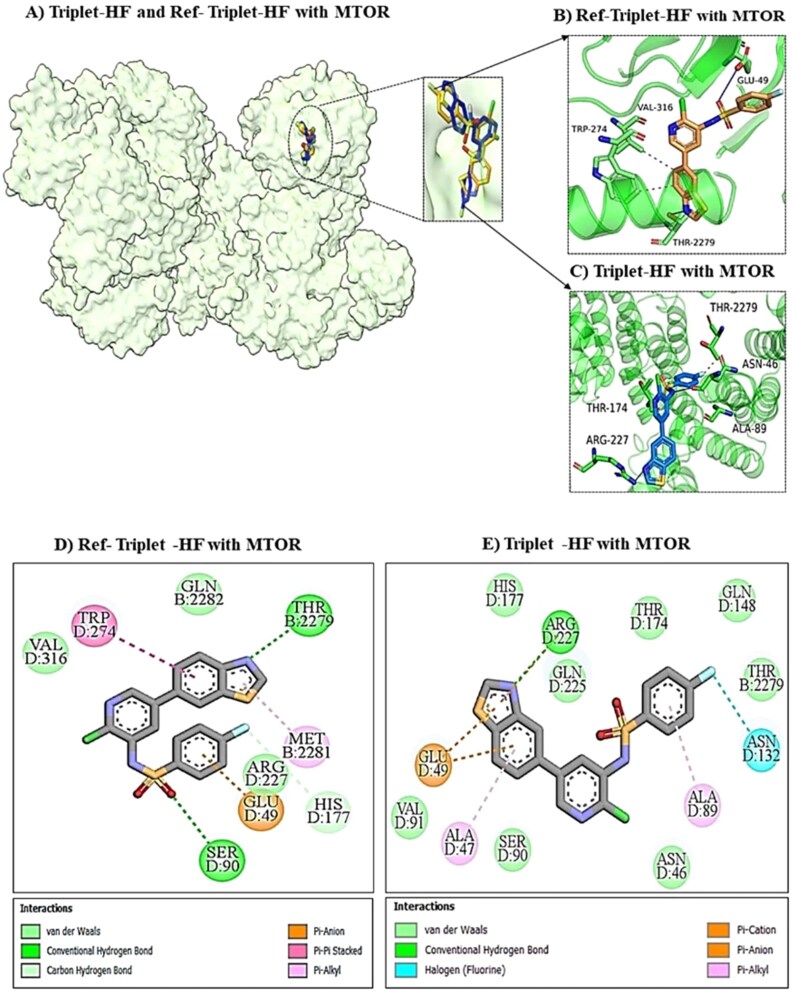
Mechanistic binding analysis of triplet-HF versus ref-triplet-HF in the mTOR active site. (A) Surface representation showing the generated triplet-HF (blue) and reference (orange) occupying the deep ATP-binding pocket of mTOR. (B and C) 3D interaction details. Triplet-HF (C) penetrates deeply into the binding cleft, engaging Thr2279 and Asn46 more effectively than the reference (B). (D and E) 2D interaction maps: a critical distinguishing feature is the formation of specific halogen bonds (cyan lines) between triplet-HF and residues Thr2279 and Asn132 (E). Combined with a denser network of van der Waals contacts (light green circles), this explains the generated candidate’s high predicted binding affinity compared to the reference, which lacks these specific halogen-mediated stabilizing interactions (D).

This dense polar network, combined with a stabilizing $\pi$-Sulfur interaction with Met227, indicates a superior geometric fit compared to the reference, which relies more heavily on $\pi$-Cation $\pi$interactions (Fig. 11D). Structurally, TripletHF engages the pocket more comprehensively, bridging the catalytic loop (Fig. 11C)in a manner distinct from the reference (Fig. 11B). In the PIK3CA active site, the ligand overlay (Fig. 11F)demonstrates that Triplet-HF targets the same hydrophobic pocket but achieves a superior interaction profile. Crucially, it forms strong hydrogen bonds with the catalytic residue Lys678 and the hinge residue Thr679, along with supporting interactions with Asn467 and Gln478 (Fig. 11J). This multi-point hydrogen bonding network, complemented by $\pi$-Cation interactions with Trp446, ensures the ligand remains firmly seated within the catalytic cleft (Fig. 11H), offering a more stable pose than the reference interaction profile, which lacks these specific anchors (Fig. 11G and I). Furthermore, in the mTOR active site (Fig. 12A), Triplet-HF distinguishes itself through specific halogen bonding. It engages Thr2279 and Asn132 via distinct halogen bonds (cyan lines) and establishes a dense network of van der Waals contacts with residues such as His177, Gln148, and Val91 (Fig. 12E).

This contrasts with the reference inhibitor, which lacks these specific halogen interactions and exhibits a less extensive contact network (Fig. 12D). The structural pose of Triplet-HF (Fig. 12C) confirms deep penetration into the binding site, supported by hydrogen bonds with Arg227 and Thr174, mechanistically explaining its favorable and balanced binding affinity across all three kinase domains. The mechanistic advantages of the generated molecules extend to the SH candidates, as demonstrated by Triplet-SH (Supplementary Fig. S16).

In AKT1, Triplet-SH maintains the essential ${\pi}$-cation interactions with the catalytic Lys179 observed in the reference but enhances affinity through a denser network of van der Waals contacts with residues such as Glu191, Leu156, and Met227 (Supplementary Fig. S16B). This broader engagement indicates a tighter geometric fit within the binding pocket compared to the reference (Supplementary Fig. S16A). Similarly, in PIK3CA, Triplet-SH introduces strong electrostatic stabilization that is absent in the reference profile. It establishes a $\pi$-Cation interaction with Lys943 and an attractive charge interaction with Glu453, supplemented by a directional halogen bond with Asp1018 (Supplementary Fig. S16D). In contrast, the reference relies primarily on weaker hydrophobic forces (Supplementary Fig. S16C). In the mTOR active site, Triplet-SH achieves a critical improvement over its reference by eliminating steric clashes. While Ref-Triplet-SH suffers from a destabilizing unfavorable donor-donor repulsion with Arg227 (Supplementary Fig. S16E, red line), Triplet-SH resolves this conflict and instead forms favorable conventional hydrogen bonds with both Arg227 and Ser90 (Supplementary Fig. S16F).

This transformation from repulsion to attraction directly correlates with its improved binding potential. These mechanistic enhancements, including mitigating steric clashes, improving shape complementarity, and establishing strong electrostatic anchoring interactions, support the model’s ability to generate novel scaffolds that are both structurally diversified and pharmacologically improved.

### Generalization study: cross-task robustness in omics-driven phenotypic drug design

The Generalization Study on Cross-Task Robustness in Omics-Driven Phenotypic Drug Design evaluated the core architecture of the Base-BiFormer-GPT model. It was applied to a distinct omics-driven drug design task through a two-phase training protocol. The first phase involved broad pre-training to establish molecular syntax, followed by targeted, correlation-driven fine-tuning in a stringent test. As a result, the model was trained based on extremely low data and noisy inputs—just 3 seed molecules per target identified via gene expression signatures, without its specialized contrastive or curriculum stages. Performance was assessed using a standardized ligand reproducibility benchmark against known drugs.

As summarized in Table 7 (Supplementary Table S7), the model demonstrated a clear competitive advantage, achieving state-of-the-art performance by attaining the highest maximum Tanimoto similarity for six of ten targets: AKT1, AKT2, CTSK, EGFR, PIK3CA, and SMAD3. This success highlights the model’s effectiveness on both inhibitory targets, such as EGFR (0.59 versus 0.386 for the next best), and activatory targets, like SMAD3 (0.58 versus 0.476). This strong performance in a mechanistically different task underscores the framework’s versatility. Experimental details, including dataset construction, transcriptomic correlation, training protocol, ligand reproducibility, and benchmarking, are described in Supplementary Information Section S8, with Supplementary Figs S17–S20.

## Discussion

This study introduces MT-ConBiFormer-GPT, a generative framework designed to tackle a critical challenge in low-data polypharmacology. A key innovation lies in its ability to generate not only dual-target but also triplet-target inhibitor candidates for the PI3K–AKT–mTOR pathway, surpassing the capabilities of many existing multi-target models that are typically limited to dual-target scenarios.

The model’s effectiveness is attributed to a three-stage training strategy integrating a sparse BiFormer encoder with a pre-trained SMILES-GPT decoder. Incorporating supervised contrastive learning is a significant advancement in this study, as it prestructures the latent space to differentiate pharmacological profiles effectively. Internal ablation studies reveal that this approach substantially enhances the performance of the MT-ConBiFormer-GPT model, enabling the generation of molecules in both dual- and triplet-target tasks with improved validity, uniqueness, diversity, and structural similarity compared to the ablated baseline, MT-BiFormer-GPT, while omitting the supervised contrastive learning stage. Demonstrating robust generalization beyond their primary training domain remains a critical challenge for generative models. To address this, we conducted two meticulous evaluations. First, the framework was benchmarked against state-of-the-art models on the DRD2/HTR1A dual-target task. In both its general and specialized H2H configurations, the model achieved competitive performance, confirming its generalizability from its primary oncology focus to neuropsychiatry.

The strong performance of the MT-ConBiFormer-GPT_H2H variant is particularly significant, as it utilizes data from the top-performing competitor, MTMol-GPT. This H2H comparison offers compelling evidence of the framework’s competitive edge and effectiveness in the multi-target molecular design domain. Second, to assess the versatility of the core architecture, we applied the foundational Base-BiFormer-GPT model, a variant without the contrastive and curriculum learning stages, to a distinct omics-driven design task.

The model outperformed state-of-the-art methods on six of ten targets in ligand reproducibility, underscoring its robustness beyond predefined target labels and its effectiveness on both inhibitory and activatory targets. The framework’s versatility stems from its synergistic architecture, pairing an efficient BiFormer encoder with a robust SMILES-GPT decoder. The encoder’s sparse attention mechanism captures long-range dependencies in SMILES with reduced computational complexity, while the pre-trained decoder fluently generates valid chemical structures. This combination of a powerful feature extractor and a robust generator creates a highly adaptable framework for diverse molecular design tasks. The pharmacological relevance of the generated molecules was confirmed through in silico validation. Top candidates, specifically the HF analog Dual-HF and Triplet-HF, achieved predicted binding affinities closely matching or surpassing those of known inhibitors. For the dual-target task, Dual-HF demonstrated superior docking scores of −9.7 kcal/mol (PIK3CA) and − 10.1 kcal/mol (AKT1), outperforming the reference mean. Similarly, Triplet-HF exhibited a robust and balanced profile across the triplet axis, with affinities of −9.4 (PIK3CA), −9.5 (AKT1), and − 10.0 (mTOR) kcal/mol.

Mechanistic analysis further validated these results, revealing that the generated ligands successfully recapitulate key binding modes, including critical hydrogen bonds with hinge residues and electrostatic interactions with catalytic sites, despite no structural refinement or optimization. This indicates the model’s intrinsic ability to generate viable therapeutic scaffolds. While results are promising, limitations remain. Binding predictions are computational and require experimental validation. Future directions include multi-objective optimization for property refinement, developing multi-modal architectures that integrate phenotypic screening data with omics signatures, and extending the model to multi-target natural product design.

In conclusion, MT-ConBiFormer-GPT establishes a powerful and adaptable framework for de novo multi-target drug design under data-scarce conditions. By uniting sparse attention, contrastive learning, and curriculum-based fine-tuning, it produces novel, valid, and diverse molecules with desirable drug-like properties. The framework demonstrates competitive performance against state-of-the-art models and generalizes effectively across both therapeutic areas and distinct design modalities.

**Key Points**

- MT-ConBiFormer-GPT is a novel deep generative framework for low-data, multi-target design. Its architecture integrates a variational autoencoder (VAE) with a BiFormer encoder and a SMILES-GPT decoder, and is optimized using a three-stage training pipeline of pre-training, contrastive learning, and curriculum learning.
- This study highlights a significant advancement by effectively producing not only dual-target but also more intricate triplet-target inhibitor candidates for the PI3K–AKT–mTOR pathway. The model demonstrates the ability to generate both High-Fidelity analog that preserve privileged pharmacophores and Scaffold-Hopping candidates that introduce novel bioisosteric cores.
- The foundational Base-BiFormer-GPT architecture confirms its versatility by achieving state-of-the-art results on a distinct omics-driven phenotypic design task.
- Mechanistic validation confirms superior binding modes. Detailed docking analysis reveals that generated candidates establish robust hydrogen, electrostatic, and halogen bonding networks with critical catalytic and hinge residues, outperforming known reference inhibitors.

## Supplementary Material

bbag079_Supplemental_Files

## Data Availability

The sample data and code for MT-ConBiFormer-GPT:https://github.com/RominaNorouzi2713/MT-ConBiFormer-GPT.git
