# Supplementary material for: MT-ConBiFormer-GPT: multi-target molecular generation for low-data drug discovery via a contrastive BiFormer-GPT architecture and curriculum learning with cross-domain generalization
Source: Brief Bioinform. 2026 May 11;27(3):bbag079. doi: 10.1093/bib/bbag079 (PMC13160422; doi:10.1093/bib/bbag079)
Supplement: bbag079_Supplemental_Files [file bbag079_supplemental_files.zip › Supplementary_Information-Revised_bbag079.docx]

**Supplementary Information for MT‑ConBiFormer‑GPT**

# Overview of Supplementary Material

This supplementary material provides a comprehensive and structured guide to the MT-ConBiFormer-GPT framework. It begins by detailing the core methodology, including the Problem Formulation (S1), the Model Architecture (S2), the Multi-stage Training Strategy (S3), Dataset Construction (S4), and the Evaluation Metrics (S5). The subsequent sections provide exhaustive validation details. Section S6 describes the experimental setup for the state-of-the-art benchmark together with the corresponding training protocol. Section S7 presents docking poses and residue-level interaction analyses for representative scaffold-hopping candidates (Figures S15–S16). Section S8 then provides a complete walkthrough of the cross-task generalization study.

# Problem Formulation

The fundamental goal of this research is the de novo generation of small molecules predicted to exhibit simultaneous activity against multiple protein targets. We formulate this as a conditional generation problem within a learned latent space, $Z$. The primary input to our model is a molecule represented by its SMILES string, which may be unlabeled or annotated with activity labels for a given multi-target profile. Our central hypothesis is that molecules sharing a desired polypharmacological profile also share learnable structural features that can be encoded into this continuous latent space. By structuring this space to reflect pharmacological relationships, we can discriminate between molecules with distinct activity profiles and generate novel, valid, and drug-like molecules by sampling from specific, well-defined regions of Z. To achieve this, we developed MT-ConBiFormer-GPT, a generative model based on a Variational Autoencoder framework. It consists of an encoder, $q_{\phi}(z\mid S)$, which maps an input SMILES string to a latent distribution, and a decoder, $p_{\psi}\left( S^{'}\mid z \right)$, which reconstructs a molecule from a sampled latent vector.

- **Input:** The primary input to our model is a molecule represented by its SMILES string, S. We work with a large chemical space of drug-like molecules, denoted by $\mathcal{M}$, such that $S\in\mathcal{M}$. For different training stages, these molecules may be unlabeled or annotated with activity labels against a set of biological targets, $T=\left\{ T_{1},\ldots,T_{k} \right\}$, where $k$ is the number of targets in a given profile (e.g., $k=2$ for dual-target, $k=3$ for triplet-target).
- **Hypothesis:** Our central hypothesis is that molecules with a desired polypharmacological profile share common, learnable structural features that can be encoded into a continuous latent space, $Z$. We posit that the geometric arrangement of this space reflects pharmacological relationships, enabling two key functions: (1) discriminating between molecules with distinct activity profiles (e.g., single- vs. multi-target), and (2) generating novel molecules with desired polypharmacology by sampling from specific, well-defined regions.
- **Output:** The desired output is a set of novel SMILES strings, $S_{new}$, that are chemically valid, unique, and possess drug-like properties. These generated molecules are sampled from the model's learned distribution and are intended to be active against a specific multi-target profile $T$.
- **Model and Objective**: To achieve this, we develop a generative model, $G_{\theta}$, based on a Variational Autoencoder (VAE) framework. The model consists of:

1. An encoder, $q_{\phi}(z\mid S)$, parameterized by $\phi$, which maps an input SMILES string $S$ to a distribution in the continuous latent space $Z$.
2. A decoder, $p_{\psi}\left( S^{'}\mid z \right)$, parameterized by $\psi$, which reconstructs a SMILES string $S^{'}$ from a sampled latent vector $z\in Z$.

# MT-ConBiFormer-GPT Architecture

| $q_{\phi}\left( z\mid S \right)\mathcal{=N}\left( \mu_{\phi}\left( S \right),\text{ }\text{diag}\left( \sigma_{\phi}^{2}\left( S \right) \right) \right)$, $p_{\psi}\left( S^{'}\mid z \right)=\prod_{t=1}^{L_{\text{max}}} p_{\psi}\left( s'_{t}\mid{S'}_{<t},z \right)$ | (1) |
| --- | --- |

The MT-ConBiFormer-GPT framework is built upon a Variational Autoencoder (VAE) architecture, which consists of two primary components:

a BiFormer-based encoder for feature extraction and a SMILES-GPT decoder for sequence generation. The overall generative process models the posterior distribution $q_{\phi}(z\mid S)$ as a Gaussian, while the decoder autoregressively generates the SMILES string $S^{'}$ from a latent vector $z$:

The following sections provide a high-level overview of each component and the multi-stage training strategy used to optimize the model.

### **Encoder**

The encoder network, $q_{\phi}(z\mid S)$, transforms an input SMILES string $S$ into the parameters ( $\mu_{\phi}(S),log\sigma_{\phi}^{2}(S)$ ) of the approximate posterior distribution. This is accomplished through a sequence of modules: This is accomplished through a sequence of modules: three 1D convolutional layers to capture local chemical motifs; the resulting features are used to construct a 2D self-relation map, creating an image-like representation; and this map is processed by our adapted BiFormer block. The core innovation of the BiFormer is its Bi-Level Routing Attention (BRA), which achieves dynamic, content-aware sparsity by first routing to a small set of relevant key-value regions before computing fine-grained attention. For a query $Q$ and routed key-value pairs $\left( K_{RQ},V_{RQ} \right)$, the attention is conceptually:

| $\text{BRA}\left( Q,K,V \right)=\text{Attention}_{\text{scaled-dot}}\left( Q,K_{R\left( Q \right)},V_{R\left( Q \right)} \right)$ | (2) |
| --- | --- |
|  |  |

This sparse attention mechanism efficiently models long-range dependencies while reducing computational complexity from the quadratic $O\left( N^{2} \right)$ of standard attention to a more scalable $O\left( N^{4}{}^{\beta} \right)$[1] . The BiFormer output is then projected through fully connected layers to produce the latent parameters, and latent vectors are sampled using the reparameterization trick to enable gradient-based optimization.

### **Decoder**

The decoder network, $p_{\psi}\left( S^{'}\mid z \right)$, reconstructs a SMILES string $S^{'}$ from a sampled latent vector $z$. The latent vector serves as a conditioning input for a pre-trained SMILES-GPT model, enabling the autoregressive generation of the output string. Subsequently, a final output layer maps the decoder's hidden states to a probability distribution over the vocabulary. This section outlines comprehensive specifications for the MT-ConBiFormer-GPT framework, encompassing layer configurations and a detailed mathematical formulation of the encoder and decoder components.

## **Encoder** Architecture Details

- **1D Convolutional Feature Extraction:** Convolutional Neural Networks (CNNs) are particularly effective at capturing local sequential patterns and structural motifs inherent in SMILES sequences. The input SMILES tensor $S\in\mathbb{R}^{N_{batch}\times L_{max}\times N_{c}}$ is processed through three sequential convolutional layers. Each layer employs learnable filters ($W_{l}^{conv}$, $b_{l}^{conv}$) followed by ReLU activations to capture increasingly abstract molecular features:

| $X^{\left( l \right)}=\text{ReLU}\left( \text{Conv1D}_{l}\left( X^{\left( l-1 \right)};W_{l}^{\mathrm{conv}},b_{l}^{\mathrm{conv}} \right) \right), l\in1,2,3$. | (3) |
| --- | --- |

These layers utilize kernel sizes of (9, 9, 11) with a stride of 1, resulting in the feature representation $X^{\left( 3 \right)}\in\mathbb{R}^{N_{batch}\times10\times L_{eff}'}$.

- **Self-Relation Matrix Construction:** To capture comprehensive pairwise feature interactions among molecular representations, the convolutional output is reshaped and converted into a 2D self-relation matrix. Specifically, the tensor $X_{conv}\in\mathbb{R}^{N\times C_{conv}\times L_{conv}}$ is permuted into $X_{conv}^{'}\in\mathbb{R}^{N\times L_{conv}\times C_{conv}}$, followed by the computation of the self-relation map:

| $X_{map}=\text{MatMul}\left( X_{conv},X_{conv}^{'} \right)$ | (4) |
| --- | --- |

This self-relation map $X_{map}\in\mathbb{R}^{N\times C_{conv}\times C_{conv}}$ is then expanded to $X_{img}\in\mathbb{R}^{N\times1\times C_{conv}\times C_{conv}}$, which serves as the input to the subsequent BiFormer attention mechanism.

- **BiFormer Attention Block:** Although BiFormer was originally developed for visual processing tasks in computer vision by Lei Zhu et al. [2023], we adapt its architecture to extract spatially structured molecular features from SMILES-based representations. This enables the model to effectively capture long-range dependencies and substructural patterns within molecular sequences. The $X_{img}$ representation is processed by a BiFormer Block module. The BiFormer architecture implements Bi-Level Routing Attention (BRA), which achieves dynamic, content-aware sparsity. For an input query $Q$, BRA first performs coarse-grained, region-level routing to identify a sparse set of $k_{top}$ (e.g., 8 in our model) relevant key-value (K-V) regions. Fine-grained token-to-token attention is then computed efficiently by restricting attention to the union of K-V tokens within these $k_{top}$ routed regions for each query token. Our BiFormer block is configured with $dim=1$, $num_{h}eads=1$, and an attention window size $n_{w}in=7$.
- **Latent Projection:** The BiFormer output is flattened and passed through a fully connected layer $fc_{0}$ (with learnable weights $W_{0}^{fc}$, $b_{0}^{fc}$ and SELU activation) to an intermediate dimension (435). This is then projected by two parallel fully connected layers, $fc_{1}$ ($W_{1}^{fc}$, $b_{1}^{fc}$) and $fc_{2}$ ($W_{2}^{fc}$, $b_{2}^{fc}$), to output the parameters of the latent distribution:

| $\mu_{\phi}\left( S \right) \text{fc}_{1}\left( \text{SELU}\left( \text{fc}_{0}\left( X_{\text{BiFormer}} \right) \right) \right),log\sigma_{\phi}^{2}\left( S \right)=\text{fc}_{2}\left( \text{SELU}\left( \text{fc}_{0}\left( X_{\text{BiFormer}} \right) \right) \right)$ | (5) |
| --- | --- |

Both $\mu_{\phi}\left( S \right)$ and $\log\sigma_{\phi}^{2}\left( S \right)$ are vectors of dimensionality $D_{z}=292.$

- **Latent Sampling:**

To facilitate effective gradient-based optimization, Latent vectors $z\in\mathbb{R}^{D_{z}}$ are sampled from $q_{\phi}\left( z|S \right)\mathcal{=N}\left( \mu_{\phi}\left( S \right),\text{diag}\left( \sigma_{\phi}^{2}\left( S \right) \right) \right)$ using the reparameterization trick:

| $z=\mu_{\phi}\left( S \right)+exp\left( 0.5\cdot log\sigma_{\phi}^{2}\left( S \right) \right)\cdot\epsilon, \epsilon\mathcal{\sim N}\left( 0,I \right)$ (6) |
| --- |

Here, $\phi$ denotes all learnable parameters of the encoder.

## Decoder **Architecture** Details

- **Latent Conditioning:** An embedding tensor derived from conditions the pretrained SMILES-GPT decoder, aligning generation with desired latent properties. The latent vector $z$ is first transformed by (with learnable weights $W_{3}^{\mathrm{fc}}$, $b_{3}^{\mathrm{fc}}$ and SELU activation). This layer projects $z$ into an embedding tensor input_embeds$\left( z \right)$ of shape $\left( L_{\max}\times D_{\mathrm{embed}} \right)$.
- **SMILES-GPT Model:** The core generative component is a pre-trained SMILES-GPT model based on the GPT-2 architecture, originally proposed by Adilov et al. [2021] for molecular language modeling. This model was trained on five million SMILES sequences from the PubChem-10M dataset using a causal autoregressive objective. Its architecture consists of 6 transformer layers, each with 12 attention heads, a hidden embedding dimension of 576, and a vocabulary size of 1072. The model was designed to capture generalizable chemical patterns, enabling its application to downstream molecular generation tasks. During each generation step $t$, the decoder predicts the next SMILES token $s'_{t}$ in a sequence $S^{'}=\left( s_{1}^{'},s_{2}^{'},\ldots,s_{L_{\max}^{'}} \right)$ autoregressively, conditioned on previously generated tokens $S'_{<t}$ and latent vector-derived embeddings. The generation process is defined as:

| $p_{\psi}\left( S^{'}\mid z \right)=\prod_{t=1}^{L_{\max}} p_{\psi}\left( s_{t}^{'}\mid S_{<t}^{'},z \right)$  $p_{\psi}\left( s_{t}^{'}\mid S_{<t}^{'},z \right)\text{= SMILES-GPT}\left( s_{t}^{'};S_{<t}^{'},\text{inputs\_embeds}\left( z \right) \right)$ | (7) |
| --- | --- |

- **Output Layer:** A final fully connected layer translates hidden states from SMILES-GPT into logits for vocabulary prediction, with a softmax providing token probabilities. For each token $s'_{t}$, the hidden state $h_{t}$ from the SMILES-GPT model (dimension $D_{\mathrm{embed}}=576$) is passed through a final fully connected layer (weights $W_{4}^{\mathrm{fc}}$, $b_{4}^{\mathrm{fc}}$). This map $h_{t}$ to logits over the output SMILES character vocabulary ($N'_{c}$). A softmax function yields the probability distribution:

| $P\left( s_{t}^{'}\mid S_{<t}^{'},z \right)=\text{Softmax}\left( fc_{4}\left( h_{t} \right) \right)$ | (8) |
| --- | --- |
|  |  |

# Inference Procedure: Multistage Training Steps

To address data scarcity and guide generation effectively, the model employs a three-phase, data-efficient learning strategy that progressively refines latent molecular representations to meet increasingly complex polypharmacological objectives. The three phases are:

1. **Unsupervised Pre-training**: A large corpus of unlabeled molecules ( $D_{\text{pretrain }}$ ) is used to pre-train the model, allowing it to acquire a general understanding of molecular syntax and semantics.
2. **Supervised Contrastive Learning:** A curated, class-balanced collection of molecules ($D_{\text{contrastive }}$) with known single-target vs. multi-target activity is used to explicitly structure the latent space, enhancing the model's ability to discriminate between pharmacological profiles.
3. **Curriculum-Based Fine-Tuning:** The model is sequentially fine-tuned, first on a specialized dataset of dual-target inhibitors ( $D_{\text{dual\_finetune }}$) and then on a dataset of triplet-target inhibitors ($D_{\text{triplet\_finetune }}$), allowing it to progressively master more complex design tasks. The following subsections provide a high-level overview of the objective function for each phase.

## Unsupervised Pretraining for Generalized Molecular Representation Learning

| $\mathcal{L}_{\mathrm{VAE}}=\mathcal{L}_{\mathrm{recon}}+\lambda_{\mathrm{KL}}\cdot\mathcal{L}_{\mathrm{KL}}$ | (7) |
| --- | --- |

In the initial phase, the VAE is trained on the large, unlabeled chemical dataset ( $D_{\text{pretrain }}$ ) to learn the fundamental syntax and semantics of SMILES strings, thereby establishing a generalizable molecular representation within a well-initialized latent space. The optimization is guided by the standard Evidence Lower Bound (ELBO), which balances reconstruction accuracy with latent space regularization:

Here, $\mathcal{L}_{\text{recon }}$ is the reconstruction loss (measured by Binary Cross-Entropy) between the input and output molecules, and $\mathcal{L}_{KL}$ is the Kullback-Leibler divergence, which encourages the learned latent distribution to approximate a standard Gaussian prior. The model was trained for a maximum of 400 epochs with a mini-batch size of 512. To maintain the pretrained language modeling capacity while enabling limited domain adaptation, all parameters were initially frozen, with the exception of the final two Transformer blocks, which were selectively unfrozen and fine-tuned. This hybrid parameter-freezing approach was integrated into the decoder initialization process. The latent vectors derived from the embedding space were decoded through a softmax-based temperature sampling mechanism, employing a fixed temperature of 1.0 to achieve an equilibrium between diversity in generation and confidence in the output. Optimization was performed with the ADOPT optimizer [2], which uses parameter-specific learning rates: a learning rate of $5\times{10}^{-4}$ was used for the fully trainable encoder, while a much lower rate of $5\times{10}^{-7}$ was used for the unfrozen decoder blocks. A uniform weight decay of $1\times{10}^{-6}$ was applied. The training objective was the VAE loss ( $\mathcal{L}_{\text{VAE }}$ ) with a Binary Cross Entropy (BCE) reconstruction term and a KL divergence scaling factor of $\lambda_{KL}=0.5$. A cosine annealing scheduler modulated learning rates, and gradient clipping (norm cap of 1.0) was used for stability. Early stopping with a patience of 30 epochs on the validation loss was implemented.

## Supervised Contrastive Learning with Hybrid Loss for Latent Space Structuring

Following pre-training, the model undergoes supervised contrastive learning to imbue the latent space with pharmacological relevance. Using the labeled dataset ( $D_{\text{contrastive }}$ ) of single-target (negative class) and multi-target (positive class) molecules, this phase explicitly teaches the model to cluster molecules with similar polypharmacological profiles. Due to the significant class imbalance, specifically the limited availability of triplet-target compounds compared to single and dual-target molecules, all dual and triplet-target instances were combined into a unified positive class during the training phase. This approach facilitated sufficient intra-class sampling, thereby stabilizing loss estimation, and improving the efficacy of contrastive representation learning. Treating triplet-target compounds as an independent class would have intensified data sparsity, thereby compromising the reliability of the learning process. To address the prevalence of false negatives in unsupervised contrastive learning, we implemented the Supervised Contrastive Learning (SupCon) framework introduced by Khosla et al.[3], which extends the InfoNCE loss to accommodate multiple positive pairs per anchor through the incorporation of ground-truth label supervision. The training objective is a hybrid loss that combines the Supervised Contrastive (SupCon) loss with a reconstruction term to maintain generative fidelity:

| $\mathcal{L}_{\text{Total}}=\mathcal{L}_{\text{SupCon}}+\mathcal{L}_{\text{Recon}}$ | (8) |
| --- | --- |

| $\mathcal{L}_{\text{SupCon}}=\sum_{i\in I} \frac{-1}{\left\vert P\left( i \right) \right\vert}\sum_{p\in P\left( i \right)} \log\frac{\exp\left( z_{i}\cdot z_{p}/\tau\right)}{\sum_{a\in A\left( i \right)} \exp\left( z_{i}\cdot z_{a}/\tau\right)}$ | (9) |
| --- | --- |

The SupCon loss, $\mathcal{L}_{\text{SupCon }}$, is defined as:

This objective pulls latent embeddings (z) from the same class together while pushing apart embeddings from different classes, creating a structured latent space that is crucial for guiding the subsequent finetuning stages. The model was initialized with weights from the pre-training phase and trained for 200 epochs with a batch size of 128. To retain the generative fidelity achieved during pretraining, we incorporated a hybrid objective that included both contrastive and reconstruction losses. The pretrained VAE decoder was retained and co-optimized alongside the encoder to preserve SMILES generation capabilities. Reconstruction accuracy was enforced through a mean squared error (MSE) loss between the input and reconstructed one-hot encoded SMILES tensors:

| $\mathcal{L}_{\text{Recon}}=\text{MSE}\left( x_{\text{recon}},x \right)$ | (10) |
| --- | --- |

This hybrid approach ensures that the latent space is influenced by both discriminative supervision and structural reconstruction constraints, producing embeddings that are both class-discriminative and chemically accurate. The model was initialized using weights obtained in the pre-training phase. The SupCon temperature parameter $\tau=0.07$ was set to 0.07. The encoder, decoder, and an additional projection head, designed as a two-layer multilayer perceptron (MLP) with ReLU activation and final L2 normalization, were optimized concurrently. Optimization was performed using the AdamW optimizer with a learning rate of $3\times{10}^{-4}$ and a weight decay of $1\times{10}^{-5}$. Upon achieving convergence, the encoder was frozen, and its latent representations were assessed using a logistic regression classifier to evaluate class separability. Metrics such as classification accuracy, confusion matrices, and two-dimensional projections of the latent space via t-SNE were employed.

## Curriculum-Based Fine-Tuning for Multitarget Generation

The fine-tuning process was implemented in two sequential phases: an initial dual-target phase focusing on PIK3CA and AKT1, followed by a more intricate triplet-target phase encompassing PIK3CA, AKT1, and MTOR. In both substages, aside from their initial parameter initialization, the models were optimized using a variational autoencoder (VAE) objective. This objective combined a binary cross-entropy (BCE) reconstruction loss with a Kullback-Leibler (KL) divergence term. A KL scaling factor ( $\beta$ ) of up to 0.05 was employed, following a linear warmup schedule (over 30 epochs in the dual-target stage and 25 epochs in the triplet-target stage), effectively balancing molecular reconstruction fidelity against latent space regularization:

| $\mathcal{L}_{VAE}=BCE\left( x_{\text{recon }},x \right)+\beta\cdot KL\left( q_{\phi}(z\mid x)\Vert p(z) \right)$ | (11) |
| --- | --- |

In Curriculum Sub-Stage A (dual-target fine-tuning), the BiFormer encoder was frozen, while fully connected layers (fc_3, fc_4), all Transformer blocks within the SMILES-GPT decoder, and the language model head were trained. During the subsequent Curriculum Sub-stage B (triplet-target fine-tuning), only the final fully connected layer (fc_4) and the language model head remained trainable, ensuring focused and efficient adaptation.

- **Curriculum Sub-stage A:** **Dual-Target Fine-Tuning (PIK3CA+AKT1):** In this initial sub-stage, the models MT-ConBiFormer-GPT and MT-BiFormer-GPT were fine-tuned on the dataset $\mathcal{D}_{\text{dual}}^{\text{finetune}}$, consisting of approximately 100 augmented SMILES strings derived from five experimentally validated dual-target inhibitors specific to PIK3CA and AKT1. Due to the small dataset size, the fine-tuning was conducted with a limited batch size of 32, optimizing generative performance specifically for dual-target molecules. The BiFormer-based encoder layers remained frozen in both variants to preserve the generalized latent representations established during pretraining. For the MT-ConBiFormer-GPT variant, initialized from supervised contrastive training, the fully connected layers (fc_3, fc_4) and all SMILES-GPT decoder components (Transformer blocks and language model head) were unfrozen for focused adaptation. Similarly, the MT-BiFormer-GPT variant, initialized from pretraining, followed an identical fine-tuning approach to facilitate direct comparative evaluation. Optimization utilized the AdamW optimizer with a learning rate of $5\times{10}^{-6}$, weight decay of $1\times{10}^{-5},KL$ weight parameter set to 0.05, and KL warmup over 30 epochs, across a maximum of 200 epochs. An early stopping mechanism with a patience of 30 epochs, based on validation loss, was implemented to avoid overfitting. Sampling employed a temperature of 0.7, top-k sampling of 20.
- **Curriculum Sub-stage B: Triplet-Target Fine-Tuning (PIK3CA+AKT1+ MTOR):** In the subsequent sub-stage, fine-tuning proceeded using the dataset $\mathcal{D}_{\text{triplet }}^{\text{finetune }}$, comprising approximately 190 augmented SMILES derived from 19 curated experimentally validated triplet-target inhibitors targeting PIK3CA, AKT1, and MTOR. Both MT-ConBiFormer-GPT and MT-BiFormer-GPT employed parameters initialized from the dual-target fine-tuning as foundational weights. Fine-tuning retained consistency in the selective layer freezing strategy, with BiFormer encoder layers and initial fully connected layers remaining frozen, while the final fully connected layer (fc_4) and SMILES-GPT language model head were selectively unfrozen for further optimization. Optimization settings included the AdamW optimizer with a learning rate of $1\times$ ${10}^{-5}$, weight decay of $1\times{10}^{-5}$, batch size of 32, KL weight parameter set to 0.05, KL warmup over 25 epochs, and early stopping with a patience of 25 epochs over a maximum of 150 epochs. Generation quality assessments occurred throughout training, using the same detailed evaluation metrics as in Sub-stage A, maintaining consistency and rigor in performance monitoring. Sampling configurations continued with a temperature of 0.7, top-k sampling of 20.

# Dataset

To facilitate the multi-stage training of our model, four distinct datasets were constructed, each aligned with a specific phase of the learning strategy: unsupervised pre-training, supervised contrastive learning, and a two-stage curriculum for fine-tuning. These datasets were compiled from public databases, including ChEMBL[4], ExCAPE-DB[5], Binding DB[6], and PubChem [7], and processed to ensure chemical validity, consistent representation, and alignment with the training objectives. The construction and purpose of each dataset in this pipeline are detailed below.

## Pretraining Dataset

To construct the pretraining dataset, we sourced and extracted compounds with high activity levels from the ChEMBL v35 database. Specifically, molecules with pChEMBL values ≥ 6 (or IC50/EC50/Kd/Ki ≤ 1 μM) were retrieved to identify bioactive compounds with a high degree of confidence. Molecular structures were standardized through the use of RDKit (version 2022.09.5), employing the canonicalization of SMILES strings, followed by the elimination of invalid entries, duplicate structures, and stereochemical information. To ensure drug-likeness, Lipinski's rule of five was applied, selecting molecules with molecular weights ranging from 200–700 Da, a maximum of five hydrogen bond donors, no more than ten hydrogen bond acceptors, and a LogP value not exceeding 5. To mitigate information leakage across training phases, molecules overlapping between the contrastive learning and fine-tuning datasets were identified. Rather than eliminating these molecules, a subset was retained and evenly distributed across the respective datasets to ensure balanced representation while reducing redundancy. Following these modifications, the finalized pretraining dataset comprised 224,243 molecules for training and 24,916 for validation, adhering to a 90/10 random split (Table S8).

**Table S8-Dataset Composition and Source Attribution Across MT‑ConBiFormer-GPT Learning Stages**

| \| **Dataset Stage Source Database(s) Training Set Size Validation Set Size** \| \| --- \| |
| --- | --- |
| Pretraining ChEMBL v35 224,243 24,916    Contrastive learning ChEMBL, ExCAPE-DB 19,427(totally) __    Stage$I$ of fine-tuning Cross-reference between:  ChEMBL, ExCAPE-DB, 90 10  (Dual Target) Binding DB, PubChem  Cross-reference between:  Stage$\mathrm{II}$of fine-tuning ChEMBL, ExCAPE-DB, 144 16  (Triplet Target) Binding DB, PubChem |

## Contrastive Learning Dataset

During the contrastive learning phase, a labeled dataset was created to facilitate the model in acquiring representations that effectively distinguish between multi-target and single-target activity within the PI3K-AKT-mTOR pathway.

- Single-Target Molecules (Negative Class, Label 0): A total of 9,867 molecules demonstrating activity against only one of the pathway targets (PIK3CA, AKT1, AKT2, or MTOR) were extracted from ChEMBL and ExCAPE-DB. After deduplication, canonicalization, and Lipinski's rule of five filtering (MW: 200–700 Da, HBD ≤ 5, HBA ≤ 10, LogP ≤ 5), they were retained as negative samples.
- Dual-Target and Triplet-Target Examples (Label 1): A total of 1,792 dual-target inhibitors and 60 triplet-target inhibitors were sourced from ChEMBL and ExCAPE-DB, exhibiting high activity with pChEMBL ≥ 6 or IC50 ≤ 1 μM) against two or more targets within the PI3K-AKT-mTOR pathway. These compounds underwent standard preprocessing, including SMILES canonicalization, deduplication, and physicochemical filtering in accordance with Lipinski's rule of five, to confirm structural integrity and drug-like properties. The distribution of multi-target data for supervised contrastive learning is illustrated in Table S9. It is noteworthy that subsequent stringent filtering was conducted, involving cross-referencing across multiple databases (ChEMBL, PubChem, ExCAPE-DB, and Binding DB), to identify chemically optimal and experimentally validated molecules for the fine-tuning phase. Given the significant class imbalance comprising 9,867 single-target molecules, 1,792 dual-target compounds, and only 60 triplet-target compounds, data augmentation through constrained SMILES randomization, while maintaining atomic connectivity and chemical validity, was implemented to mitigate this issue [8]. This augmentation increased the dual-target set fivefold and the triplet-target set tenfold, yielding a combined multi-target class of 9,560 molecules. This approach effectively balanced the class distribution between the 9,867 single-target (negative class) and augmented multi-target (positive class) molecules, facilitating more balanced and efficient supervised contrastive learning. The final contrastive dataset, comprising 19,427 molecules, enabled structured latent space training for distinguishing single-target from multi-target polypharmacological profiles.

**Table S9- Distribution of multitarget inhibitors across dual and triplet-target combinations within the PI3K-AKT-mTOR pathway before filtering and dataset refinement for fine-tuning**.

| Target Combination | Molecule Count |
| --- | --- |
| **Dual-Target Inhibitors** |  |
| PIK3CA + MTOR | 1,540 |
| AKT1 + AKT2 | 213 |
| AKT1 + MTOR | 11 |
| PIK3CA + AKT1 | 26 |
| PIK3CA + AKT2 | 2 |
| **Triplet-Target Inhibitors** |  |
| PIK3CA + AKT1 + MTOR | 55 |
| AKT1 + AKT2 + MTOR | 4 |
| PIK3CA + AKT1 + AKT2 | 1 |

## Fine-Tuning Datasets

In the context of curriculum-based fine-tuning, we developed two domain-specific datasets that align with the sequential training strategy.

- $\mathcal{D}_{\text{dual}}^{\text{finetune}}$ : Used for Stage I fine-tuning to generate dual-target inhibitors against the PIK3CA/AKT1 combination.
- $\mathcal{D}_{\text{triplet}}^{\text{finetune}}$ : Used for Stage II fine-tuning to generate triplet-target inhibitors for the PIK3CA/AKT1/MTOR combination.

The fine-tuning subsets were generated from dual and triplet-target molecules initially collected for contrastive learning. These compounds were chosen based on their high biological activity (pChEMBL ≥ 6 or IC50 ≤ 1 μM) and obtained from ChEMBL and ExCAPE-DB. A standardized preprocessing workflow was implemented, encompassing SMILES canonicalization, deduplication, and filtering according to Lipinski’s criteria for drug-likeness.

To isolate chemically optimal compounds, the following physicochemical criteria were applied:

- **Dual-target set**: MW 300–450 Da; LogP 2–4; HBD ≤ 3; HBA 2–7
- **Triplet-target set**: MW 350–500 Da; LogP 2.5–4.5; HBD ≤ 3; HBA 3–8

Based on these constraints, the PIK3CA+AKT1 combination was selected from the dual-target subset, yielding 5 high-quality molecules with median MW ~350–370 Da and LogP 3.2–3.6. From the triplet-target subset, the PIK3CA+AKT1+MTOR combination provided 19 molecules, despite their slightly higher MW (~440–480 Da), with acceptable LogP, HBD, and HBA values. These combinations were chosen over others due to their favorable physicochemical profiles, as shown in Supplementary Figures S1,2.

To ensure a reliable generative process, only molecules with experimentally verified dual- or triplet-target binding profiles were retained. These profiles were confirmed by cross-referencing a number of databases, including ChEMBL, PubChem, ExCAPE-DB, and Binding DB. As a result of this rigorous filtering, not only were the selected compounds chemically and pharmacologically appropriate, but also had consistent multi-source target annotations, thus preserving biological fidelity in the generative model. In response to the limited size of the fine-tuning data, SMILES randomization was conducted using a constrained atom-order protocol. The 5 dual-target molecules were augmented 20-fold and the 16 triplet-target molecules 10-fold, resulting in 100 and 160 molecules, respectively (Table S9). The procedure ensured that the enriched datasets retained the same physicochemical distributions as the original data, as confirmed through comparisons of LogP, HBD, HBA, and molecular weight, along with Tanimoto similarity analysis (Supplementary Figures S3,4). Following data augmentation, the resulting datasets were partitioned into 90% training and 10% validation subsets, resulting in 90/10 molecules for the dual-target set and 144/16 molecules for the triplet-target set. The full set of charge-colored two-dimensional structures for all five dual-target and sixteen triplet-target ligands before undergoing augmentation is available in Supplementary Figures S5 and S6, respectively.


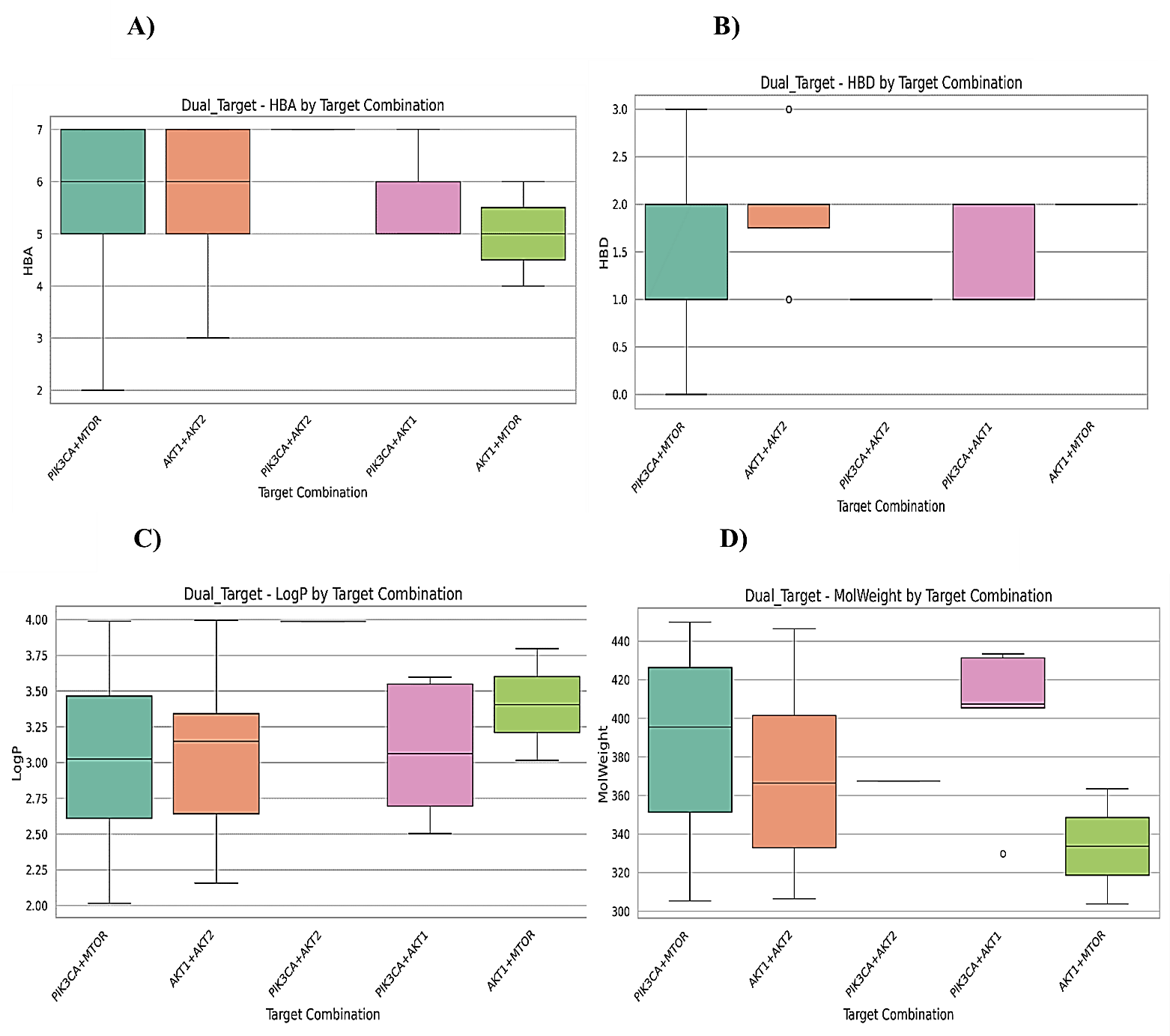


**FigureS1.** **Box plots illustrating physicochemical property distributions of dual-target combinations extracted from ChEMBL and ExCAPE-DB**.

**(A)** Hydrogen bond acceptors (HBA),

(**B)** Hydrogen bond donors (HBD),

**(C)** LogP, **(D)** Molecular weight (MolWeight). Among the analyzed combinations, PIK3CA+AKT1 compounds exhibit the most favorable drug-likeness profiles: MolWeight ~ MW ~350–370 Da, LogP 3.2–3.6, HBD ≤ 3; HBA 2–7, guiding their selection as the primary dual-target fine-tuning subset.

**
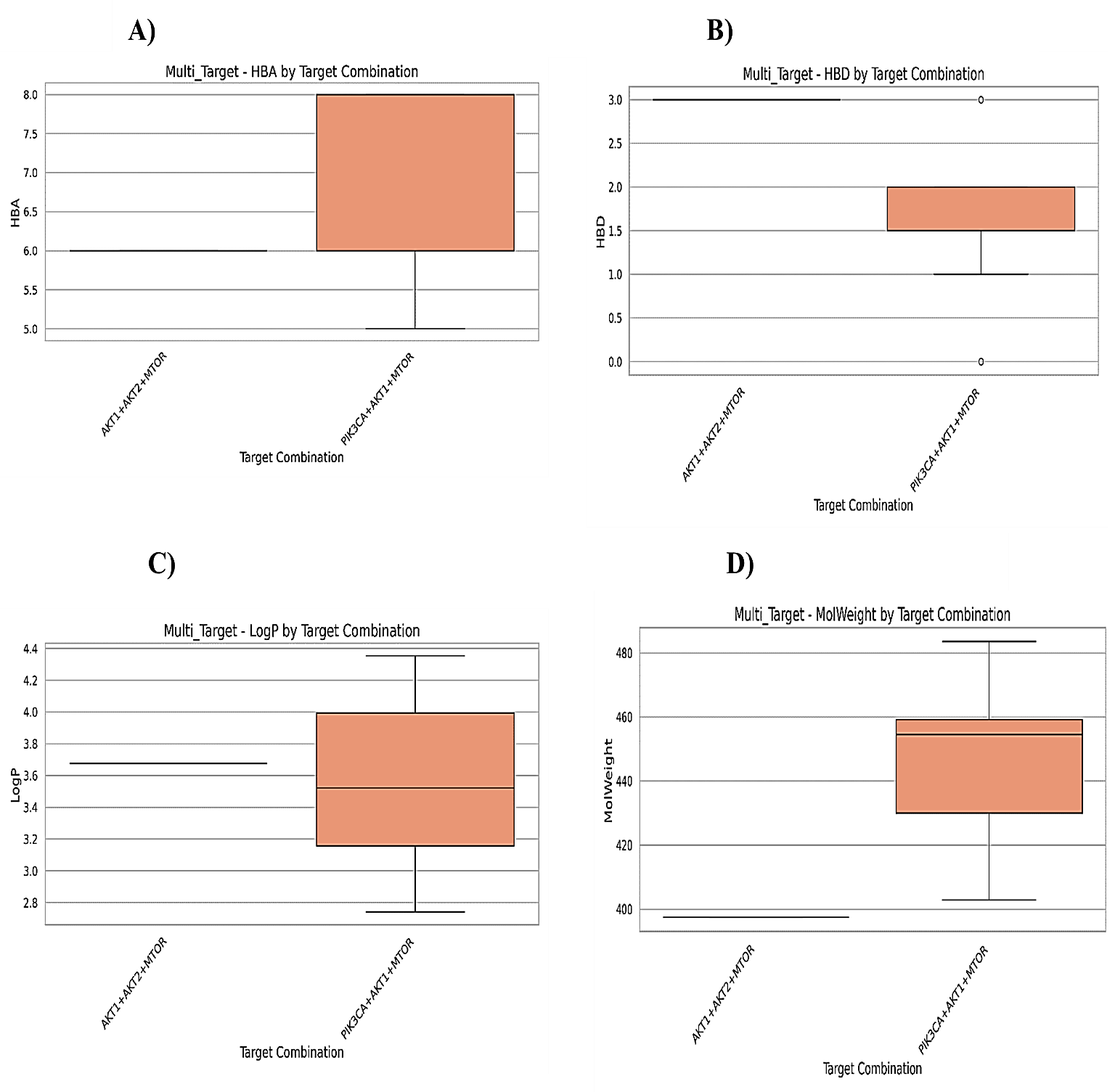
**

**FigureS2. Box plots illustrating physicochemical property distributions of triplet-target combinations extracted from ChEMBL and ExCAPE-DB.**

**(A)** Hydrogen bond acceptors (HBA),

**(B)** Hydrogen bond donors (HBD),

**(C)** LogP, **(D**) Molecular weight (MolWeight). Among the analyzed combinations, PIK3CA+AKT1+MTOR compounds exhibit the most favorable drug-likeness profiles—MolWeight ~440–480 Da, LogP between 3.2–4.2, HBD ≤ 3, and HBA within 5–8—guiding their selection as the primary triplet-target fine-tuning subset.

**
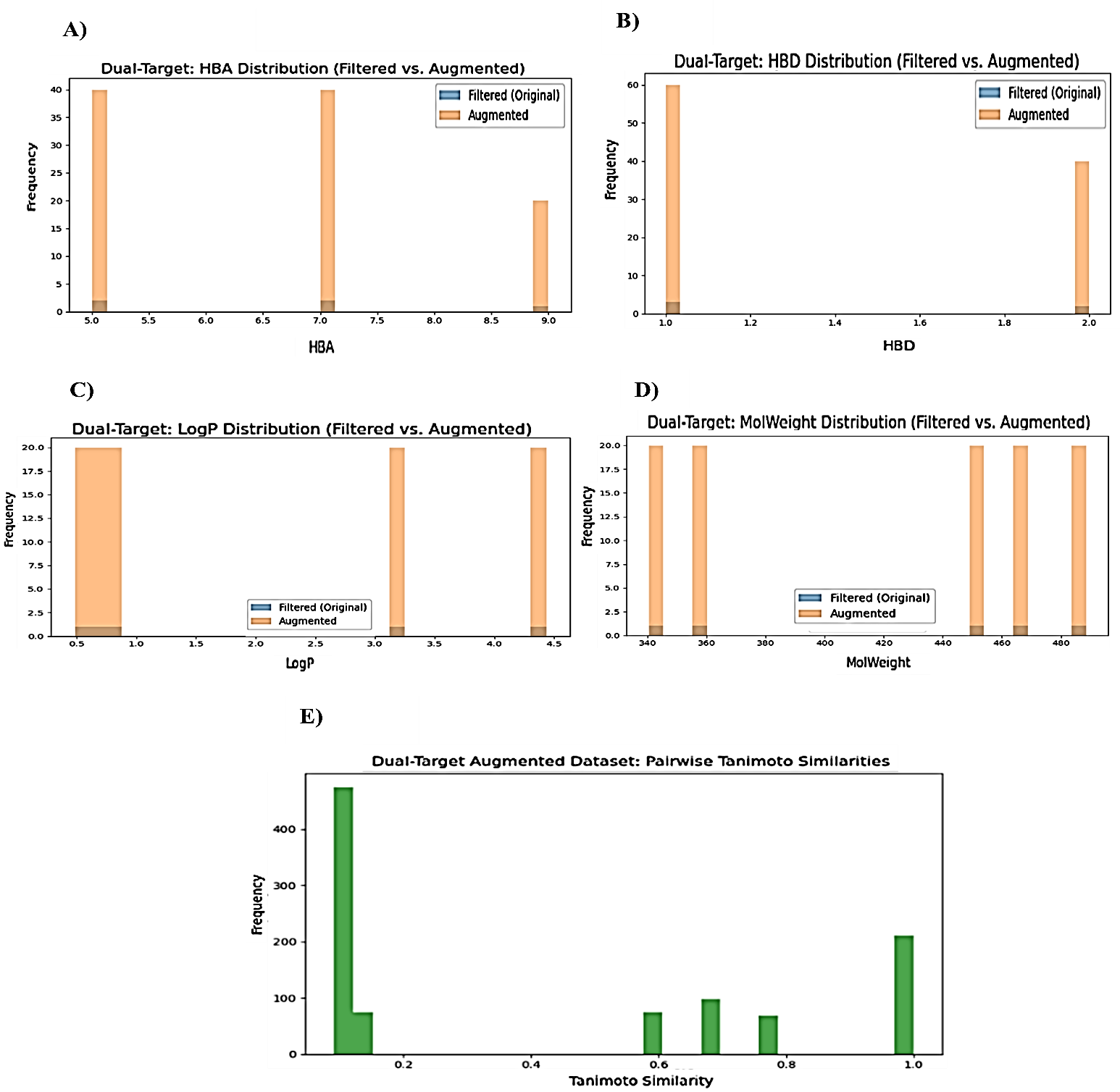
**

**FigureS3. Physicochemical property comparison between original (filtered) and augmented dual-target datasets.**

**(A)** Distribution of hydrogen bond acceptors (HBA).

**(B)** Distribution of hydrogen bond donors (HBD).

**(C)** Distribution of logP values.

(**D)** Distribution of molecular weight.

**(E)** Tanimoto similarity analysis for the augmented dual-target molecules.

**
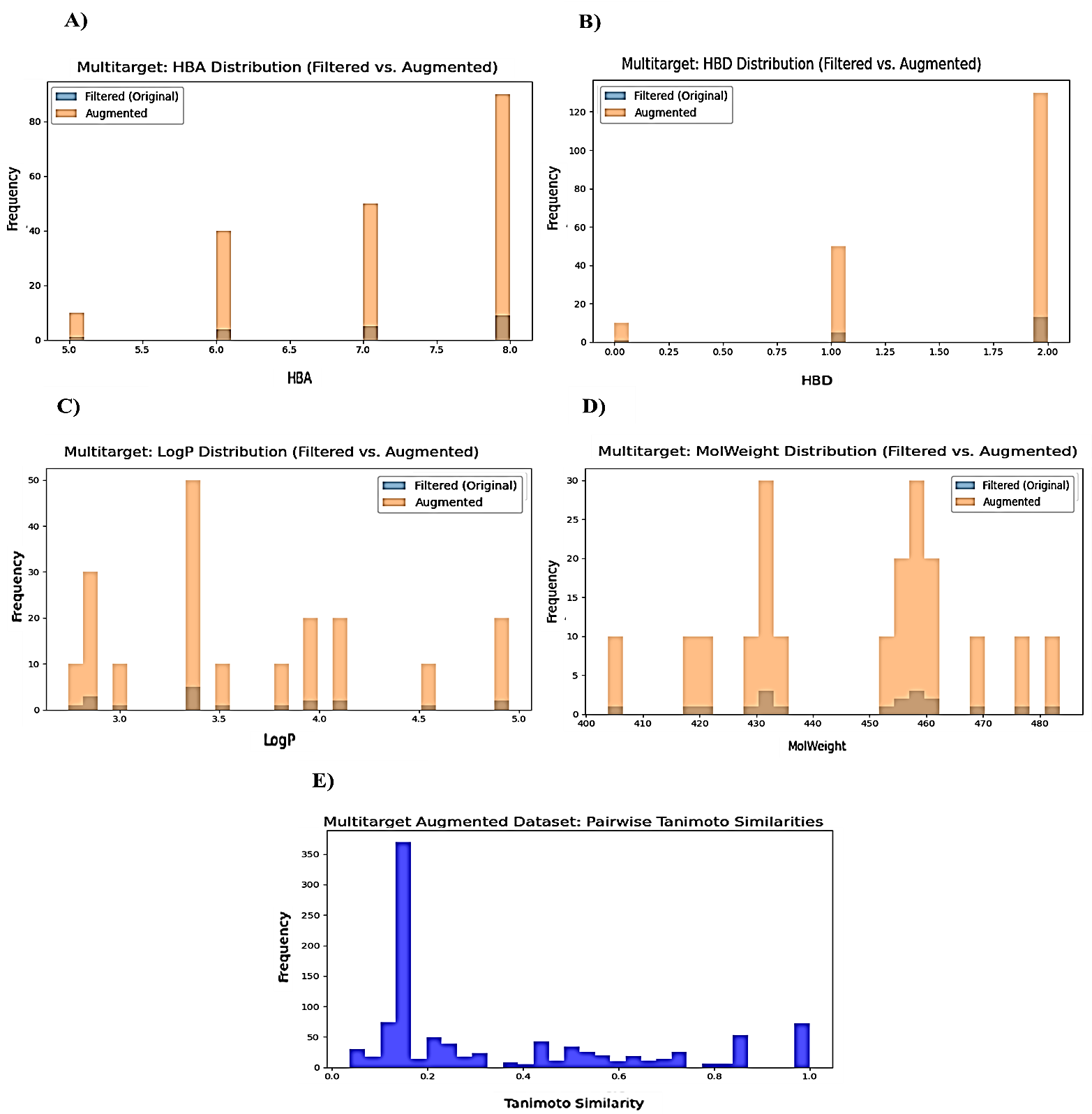
**

**FigureS4**. Physicochemical and structural similarity analysis of the augmented triplet-target dataset**. (A)** Distribution of hydrogen bond acceptors (HBA) before and after augmentation. **(B)** Distribution of hydrogen bond donors (HBD). **(C)** LogP distribution. **(D)** Molecular weight (MolWeight) distribution. **(E)** Tanimoto similarity among augmented triplet-target molecules, indicating the chemical diversity retained during augmentation. These analyses confirm that the augmentation preserved the original physicochemical properties.


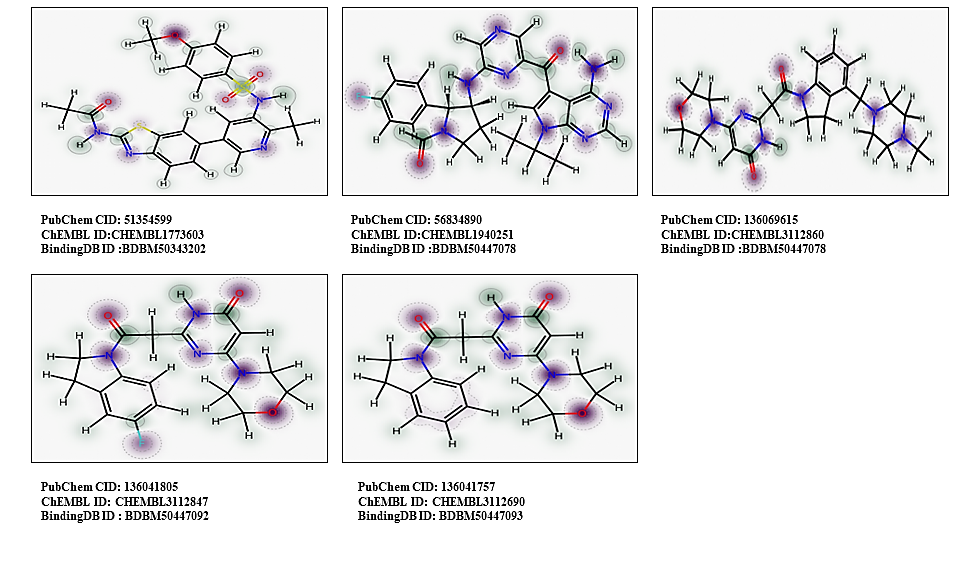


**Figure S5. Charge-mapped two-dimensional structures of the five dual-target reference ligands (PIK3CA + AKT1). Halos show Gasteiger partial charges rendered with the PRGn palette (purple ↔ green); eight dotted contour levels, α = 0.7.**


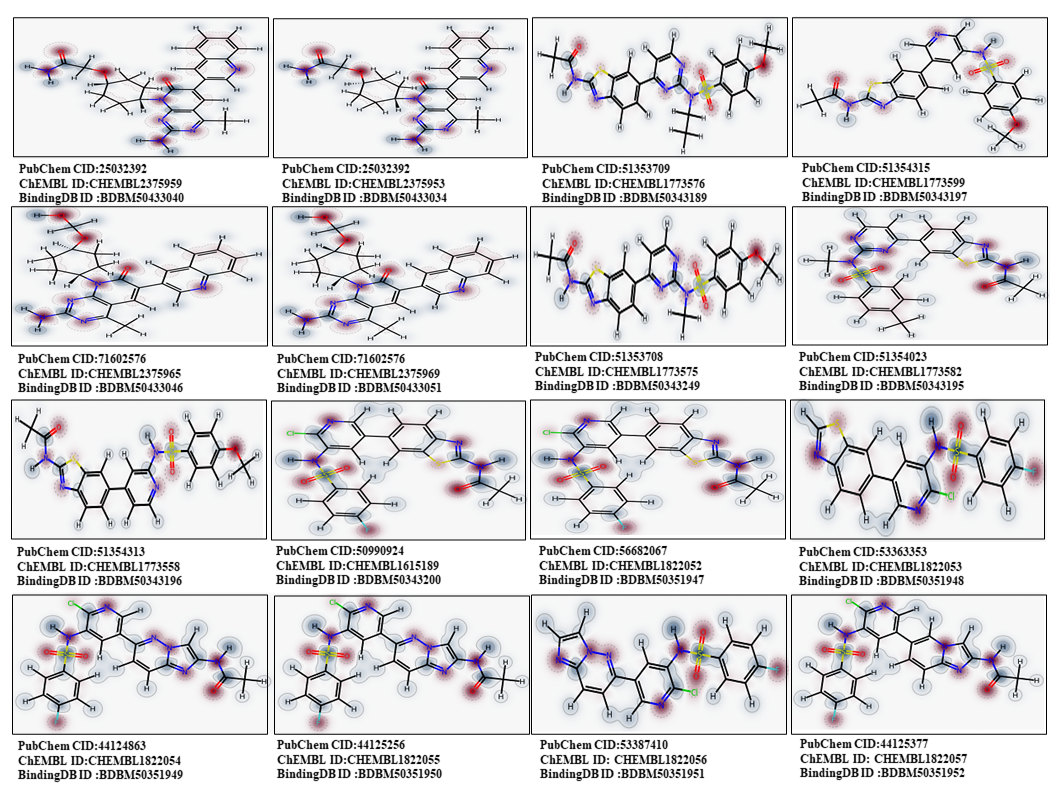


**Figure S6. Charge-mapped two-dimensional structures of the sixteen triplet-target reference ligands (PIK3CA + AKT1 + MTOR). Halos show Gasteiger partial charges rendered with the RdBu palette (red ↔ blue); eight dotted contour levels, α = 0.7.**


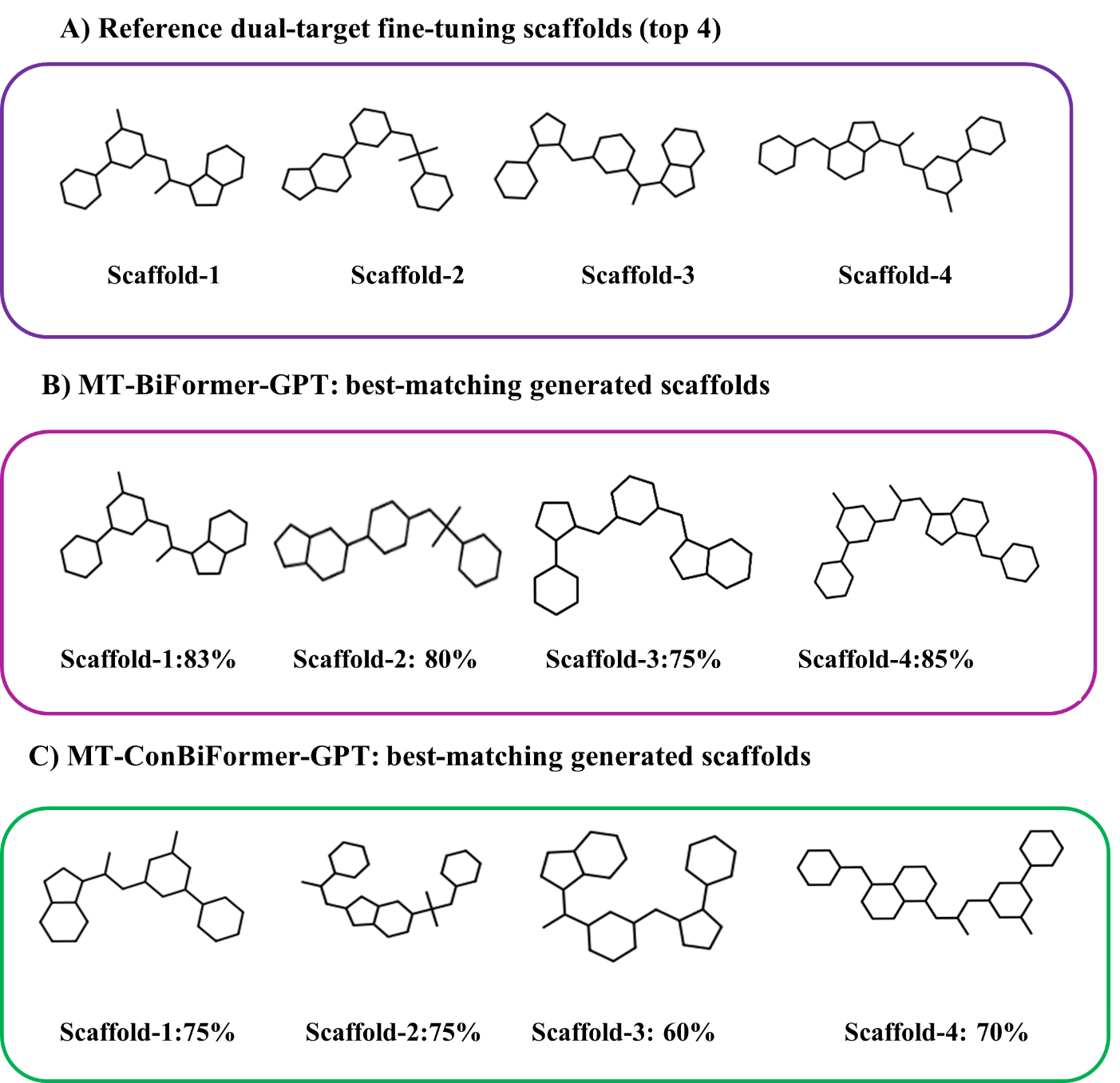


**Figure S7-Scaffold recovery and scaffold hopping for dual-target generation**. **A)** four most frequent Murcko scaffolds in the PIK3CA–AKT1 dual-target fine-tuning set (Scaffold-1–4). **B)** best-matching dual-target scaffolds generated by the ablated baseline MT-BiFormer-GPT.**C)** best-matching dual-target scaffolds generated by the full MT-ConBiFormer-GPT model. The percentages under each structure report the Murcko-based Tanimoto similarity between the generated scaffold and its corresponding reference scaffold. MT-BiFormer-GPT mainly regenerates the training scaffolds with very high similarity, whereas MT-ConBiFormer-GPT retains the same cores but introduces more extensive modifications to the periphery, consistent with scaffold hopping around known dual-target chemotypes.


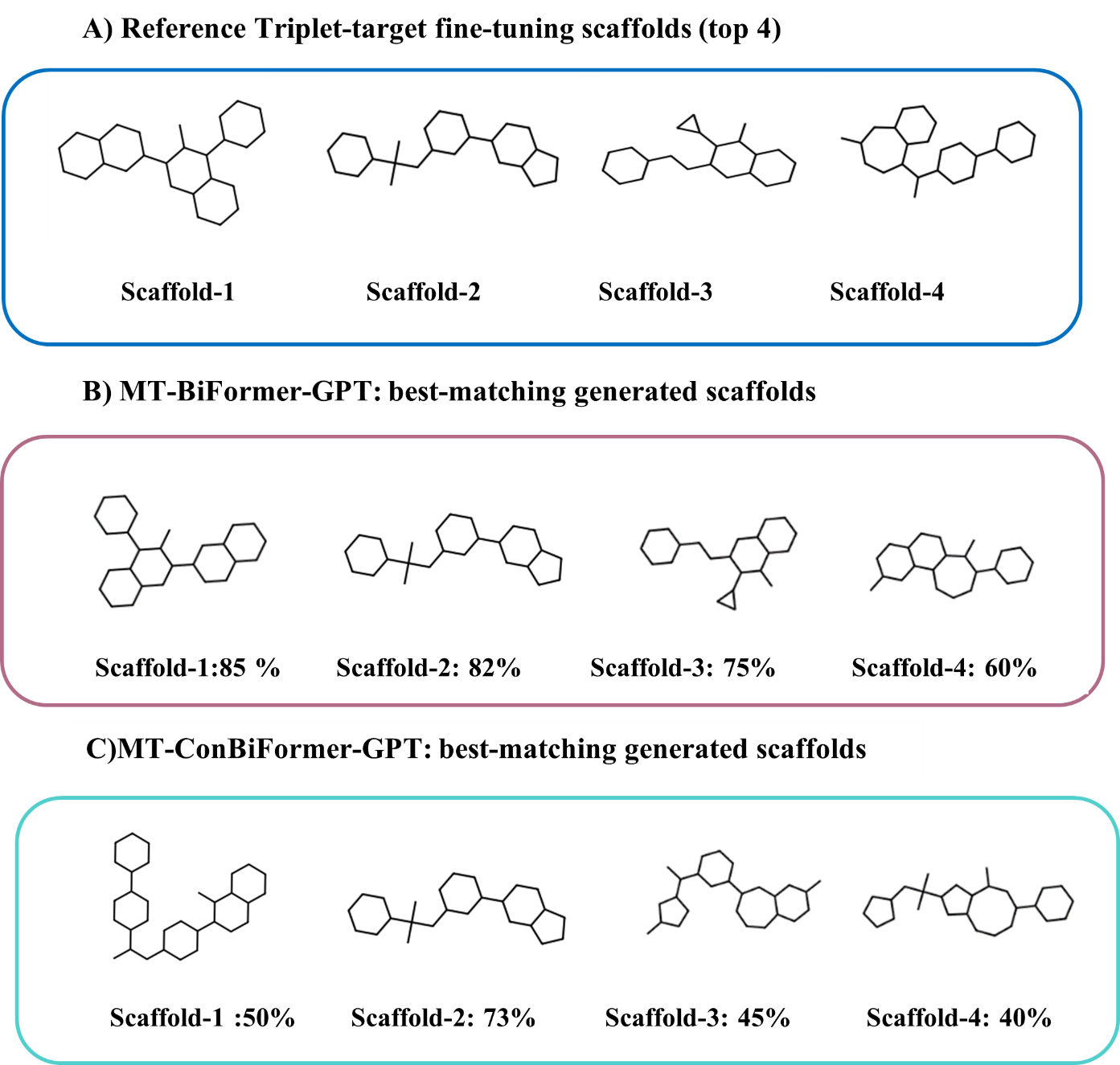


**Figure S8-Scaffold recovery and scaffold hopping for triplet-target generation.** **A)** four most frequent Murcko scaffolds in the PIK3CA–AKT1–MTOR triplet-target fine-tuning set. **B)** best-matching triplet-target scaffolds generated by MT-BiFormer-GPT. **C)** best-matching triplet-target scaffolds generated by MT-ConBiFormer-GPT. Percentages indicate Murcko-based Tanimoto similarity to the corresponding reference scaffold. Both models can reconstruct the complex fused-ring and sulfonamide chemotypes present in the training data, but MT-ConBiFormer-GPT preferentially generates analogues with lower scaffold similarity, reflecting increased scaffold diversity while preserving key multi-target pharmacophoric features.

# Evaluation metrics

To rigorously assess the generative capabilities and pharmacological relevance of MT-ConBiFormer-GPT, a comprehensive set of evaluation metrics was implemented. These metrics adhere to established methodologies in the de novo molecular generation literature, with a particular focus on benchmarking multi-target approaches [9, 10]. The evaluation included structural validity, chemical uniqueness and novelty, internal chemical diversity, target-specific structural similarity (utilizing the MOSES benchmark), physicochemical properties indicative of drug-likeness, and molecular docking simulations to estimate binding affinities. This holistic methodology enabled a thorough evaluation of both the chemical integrity and the biological relevance of the generated molecules.

- - 1. **Structural Validity, Uniqueness, and Novelty**

The quality of the generated molecules was evaluated based on three primary metrics, aligned with the standards of the MOSES [11] benchmarking platform:

- Validity: Defined as the percentage of generated SMILES strings that conform to chemical validity criteria as determined by RDKit [12], this metric evaluates the model's ability to generate molecular structures that are both syntactically and semantically correct.
- uniqueness: Unique@K quantifies the proportion of unique molecules within the first K valid molecules generated. As defined by the MOSES benchmark, this metric was calculated for K = 1,000 and K = 10,000 valid molecules, serving as an indicator of the model's capacity to produce diverse and non-repetitive molecular structures.
- Novelty: this metrics is defined as the proportion of unique, valid generated molecules absent from the combined training datasets (pretraining, contrastive, and fine-tuned sets). This metric underscores the model's ability to achieve authentic de novo generation, distinct from mere replication of training data.
  - 1. **Internal Chemical Diversity**

The chemical diversity within the set of valid and unique generated molecules was assessed to evaluate the variability in the generated library. Ensuring high internal diversity is essential for comprehensive exploration of the chemical space and mitigating the risk of model collapse toward a restricted set of structures. This evaluation was conducted by calculating the average pairwise Tanimoto similarity using Morgan fingerprints (e.g., radius 2, 2048 bits) for the generated molecules. A lower average similarity corresponds to greater diversity.

- - 1. **Target-Specific Structural Similarity**

To evaluate the structural similarity between the generated molecules and known bioactive compounds targeting the PI3K-AKT-mTOR pathway, several metrics from the MOSES (Molecular Sets) benchmarking framework were applied. The reference set for these comparisons comprised known active molecules for PIK3CA, AKT1, and MTOR. These metrics include:

- **The Fréchet ChemNet Distance (FCD):** FCD is a metric designed to assess the similarity between the distributions of generated and reference molecules by leveraging features extracted from a pre-trained deep neural network, ChemNet. This approach captures similarities in both chemical structure and predicted bioactivities. Notably, lower FCD values correspond to higher levels of similarity [13].
- **Similarity to Nearest Neighbor (SNN):** Calculated as the average Tanimoto similarity (using Morgan fingerprints) between each generated molecule and its closest counterpart in the reference set of known active molecules. Higher SNN values suggest that generated molecules are structurally close to known actives.
  - 1. **Physicochemical and Drug-Likeness Properties**

To ensure the generated molecules exhibit characteristics suitable for drug development, a comprehensive analysis of key physicochemical properties was conducted, including the following:

- **Quantitative Estimate of Drug-Likeness (QED):** A score from 0 to 1 that reflects the aesthetic appeal of a molecule from a medicinal chemistry perspective [14].
- **Synthetic Accessibility (SA) Score:** A quantitative measure of the relative ease with which a molecule can be synthesized, with values typically ranging from 1 (indicating high feasibility) to 10 (indicating significant complexity)[15].
- **Partition Coefficient (LogP**): The partition coefficient (LogP) represents the logarithm of the n-octanol/water distribution ratio, serving as a measure of a molecule's lipophilicity. The property distributions of the generated molecules were evaluated relative to established drug-likeness benchmarks, such as Lipinski's rule of five and, where applicable, compared to the distributions of known drugs or the training dataset [16].
- **Scaffold Diversity: A** critical measure of a generative model's effectiveness is its ability to produce structurally novel molecules rather than merely reproducing training data.

# State-of-the-Art Benchmark Experimental Details

This section outlines the comprehensive methodology, dataset construction, and intermediate findings of the state-of-the-art (SOTA) benchmark study. The objective was to evaluate MT-ConBiFormer-GPT by comparing its performance against leading multi-target generative models on the standard DRD2/HTR1A dual-target task.

## Benchmark Dataset Preparation

For both general and Head-to-Head (H2H) benchmarks, datasets are prepared for pretraining, contrastive learning, and fine-tuning.

### **General and Head-to-Head Benchmark Data Pretraining Construction**

The ChEMBL dataset, comprising 344,184 bioactive molecules, has been employed by state-of-the-art models such as DLGN and MTMol-GPT for pretraining in benchmark evaluations.

### **Head-to-Head (H2H) Benchmark Dataset Contrastive Learning and Fine-tunning Construction**

To enable the most rigorous and direct comparison with the top-performing competitor, MTMol-GPT, a dedicated Head-to-Head (H2H) dataset was developed, utilizing their precise data sources.

- Contrastive Learning set: Negative Class (Single-Target): The negative class (Label 0) was compiled directly from the training files used in the MTMol-GPT study. This includes 1,293 unique SMILES for the DRD2 target and 1,672 unique SMILES for the HTR1A target, resulting in a total of 2,965 unique single-target molecules. Positive Class (Dual-Target): The positive class (Label 1) was derived from a publicly available set of 1,000 candidate molecules from the MTMol-GPT study, for which predicted binding affinities against both DRD2 and HTR1A targets were provided. To create a high-confidence set, we filtered these molecules, retaining only those with a strong predicted binding affinity of < -7.0 kcal/mol for both targets. This process yielded 937 high-quality dual-target molecules. To creates a class-balanced dataset for the supervised contrastive learning stage, the 937 dual-target molecules were augmented 3-fold using constrained SMILES randomization. The final dataset for H2H contrastive learning consisted of 2,965 single-target molecules (Label 0) and 2,811 augmented dual-target molecules (Label 1).
- Fine-tunning set: For the fine-tuning stage, the dataset was comprised of the 937 original dual-target molecules combined with the 2,811 augmented SMILES that were used for contrastive learning to guide the generator toward the DRD2/HTR1A chemical space.

### **General Benchmark Dataset Contrastive Learning and Fine-tunning Construction**

DRD2 and HTR1A molecules sourced from the ExCAPE-DB database were used for the general comparison against all SOTA models. The initial dataset consisted of 5,006 single-target DRD2 molecules, 4,276 single-target HTR1A molecules, and 1,399 dual-target DRD2/HTR1A molecules.

- Contrastive Learning set: The single-target DRD2 and HTR1A files were merged to create the negative class (Label 0). The dual-target molecules were augmented 5-fold to create a balanced positive class (Label 1). The final dataset for general contrastive learning comprised 8,872 unique single-target molecules and 6,930 augmented dual-target molecules.
- The fine-tuning stage of the general benchmark utilized the original, un-augmented dataset comprising 1,399 dual-target molecules.

## Benchmark Training Protocol

The benchmark protocol was structured in three phases: (1) unsupervised pre-training, (2) supervised contrastive learning, and (3) fine-tuning, applied to both the general and Head-to-Head benchmarks.

### **Unsupervised Pre-training**

The objective of this phase was to allow the model to learn the fundamental syntax of SMILES strings. The model was trained for a maximum of 1000 epochs with a batch size of 512, using the ADOPT optimizer with distinct learning rates for the VAE ($5\times{10}^{-4})$ and the unfrozen SMILES-GPT layers ($5\times{10}^{-7})$. A CosineAnnealingLR scheduler, a KL divergence weight of 0.5, and an early stopping patience of 30 epochs were employed. The model converged successfully, as shown by the training loss curve in Supplementary Figure S9.

### **Supervised Contrastive Learning**

After the pre-training phase, the model underwent fine-tuning for 200 epochs on benchmark datasets to organize the latent space according to class labels. This supervised training phase employed a batch size of 128 and the AdamW optimizer, with a learning rate of $3\times{10}^{-4}$and a weight decay of$1\times{10}^{-5}$. The optimization was guided by a hybrid loss function, which combined the Supervised Contrastive (SupCon) loss (with a temperature parameter τ=0.07) and a Mean Squared Error (MSE) reconstruction loss to maintain generative fidelity. The training convergence and confusion matrix for the General Benchmark are presented in Supplementary Figure S10, with corresponding results for the H2H Benchmark available in Supplementary Figure S11.


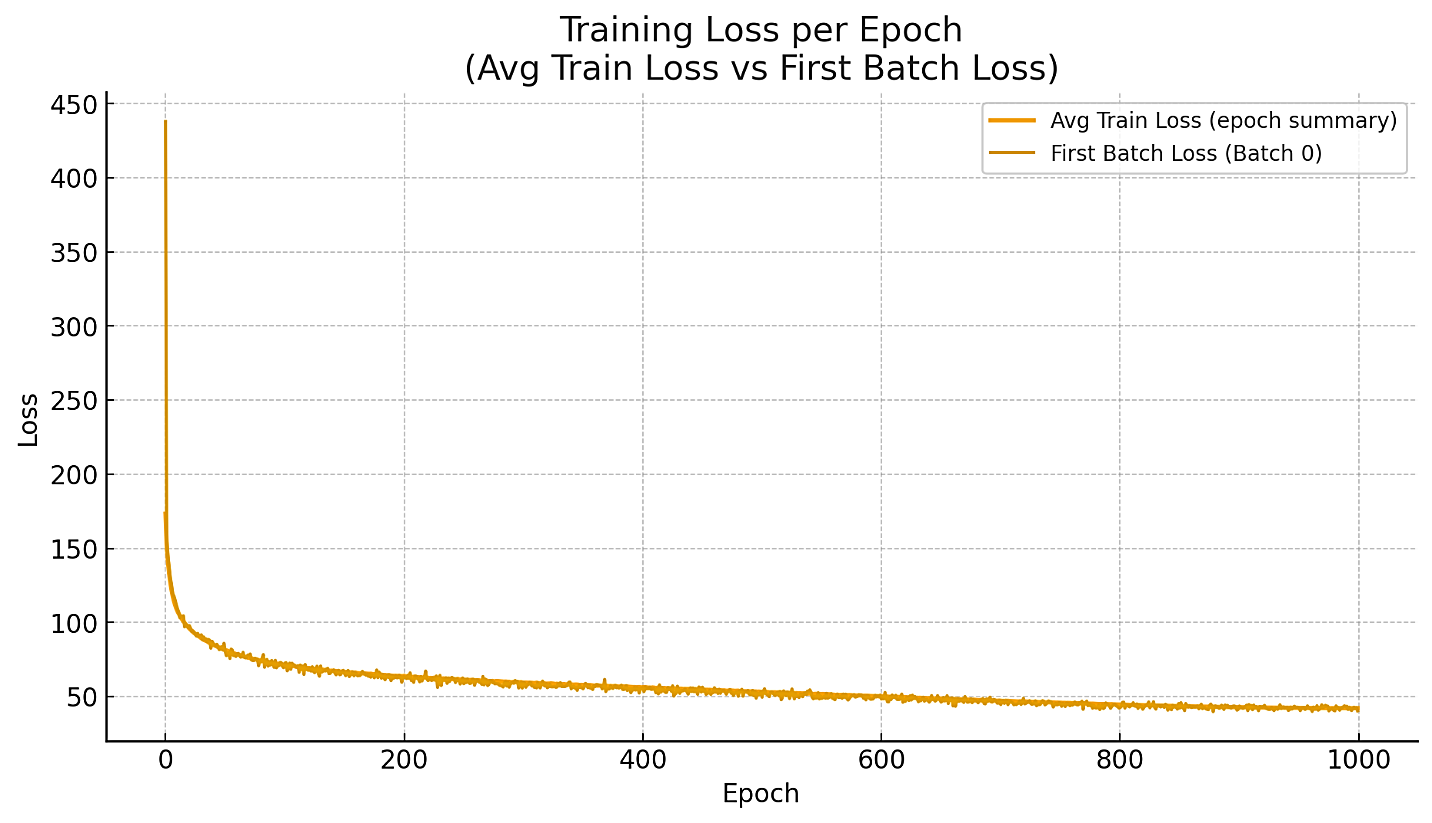


**FigureS9. Training loss curve for the unsupervised pre-training phase on the ChEMBL dataset for the SOTA benchmark.**


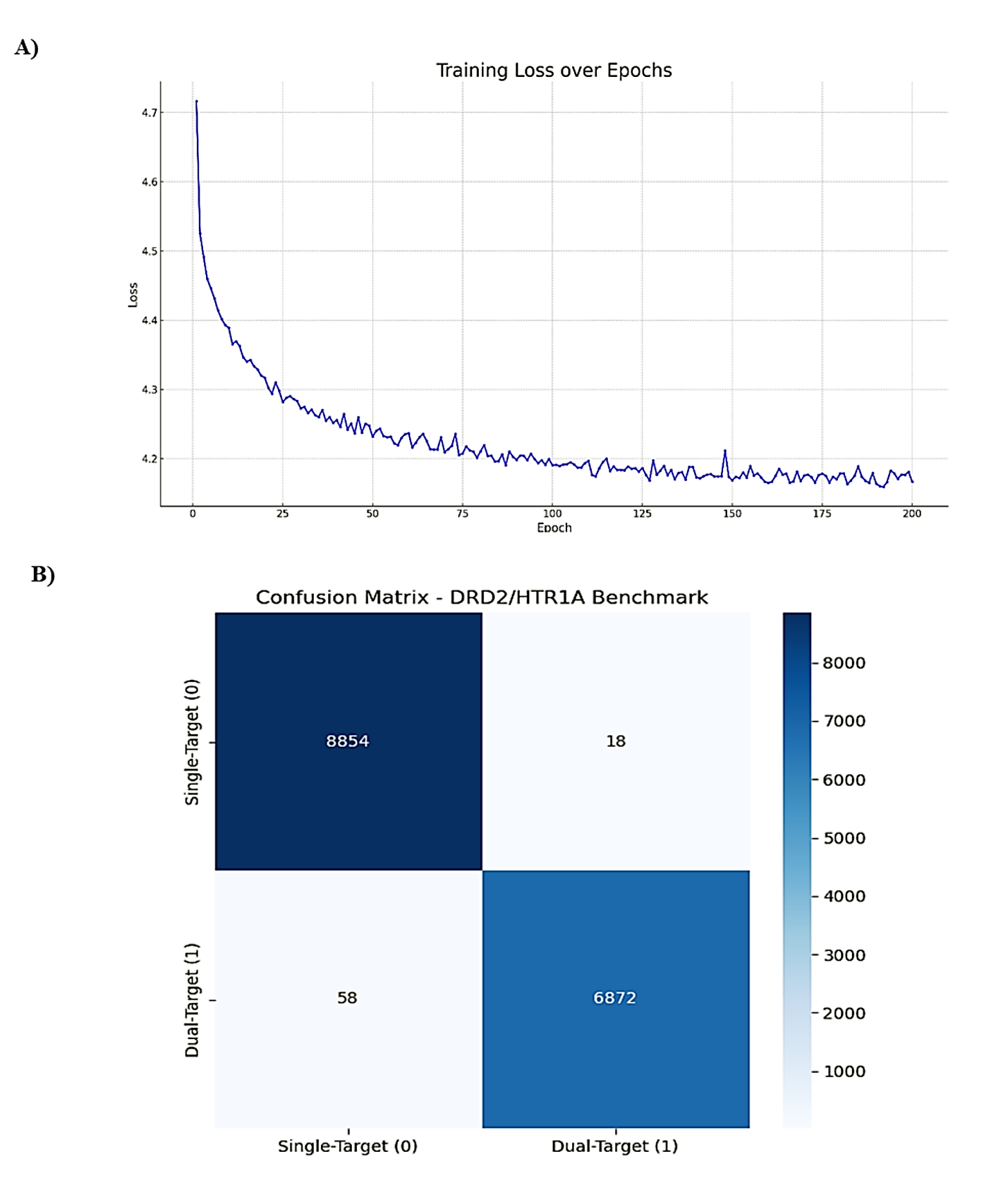


**Figure S10. Performance of Supervised Contrastive Learning on the General Benchmark. (A)** The training loss curve for the hybrid Supervised Contrastive (SupCon) and MSE reconstruction loss, plotted over 200 epochs. (B) A confusion matrix from a logistic regression classifier trained on the final latent space representations.


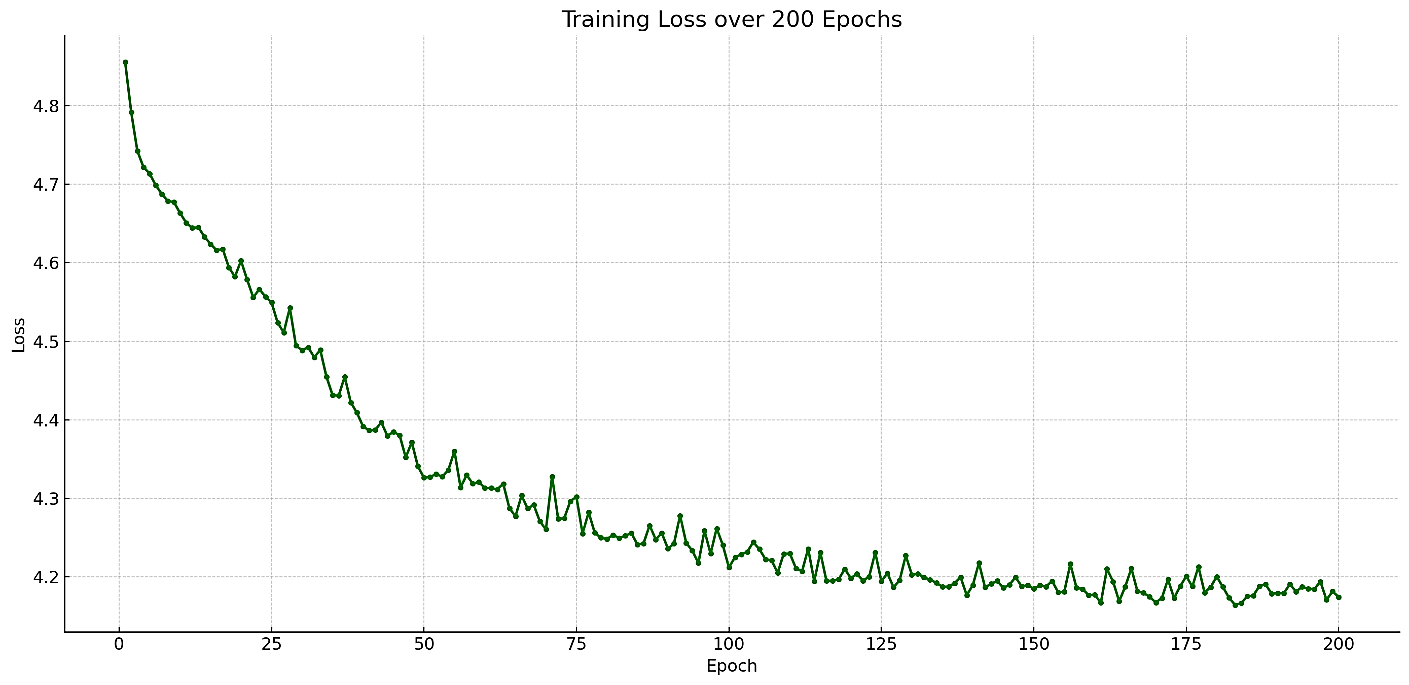

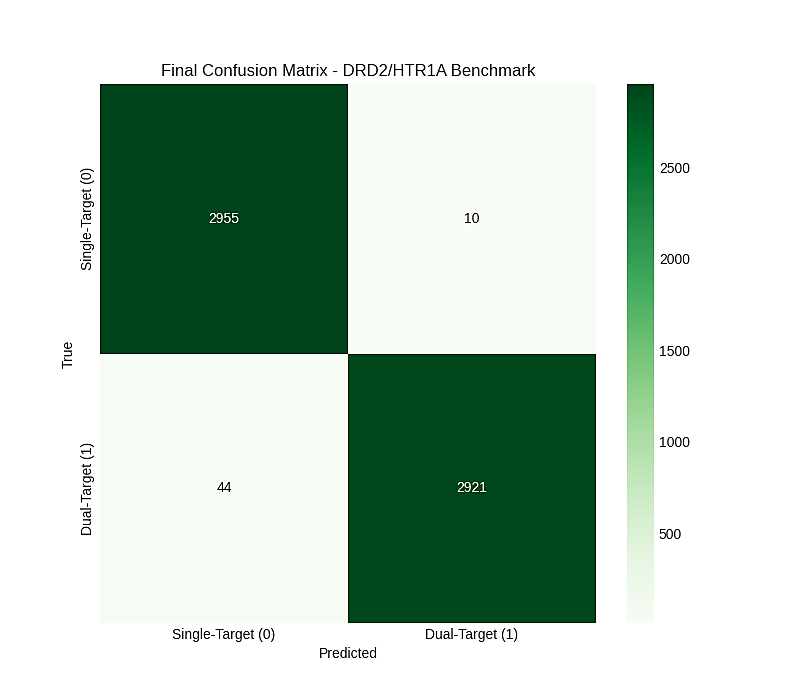


**A)**

**B)**

**Supplementary Figure S11: Performance of Supervised Contrastive Learning on the Head-to-Head (H2H) Benchmark**. **(A)** The training loss curve for the hybrid Supervised Contrastive (SupCon) and Mean Squared Error (MSE) reconstruction loss, depicted over 200 epochs. **(B)** A confusion matrix generated from a logistic regression classifier trained on the final latent space representations.

### **Fine-tuning**


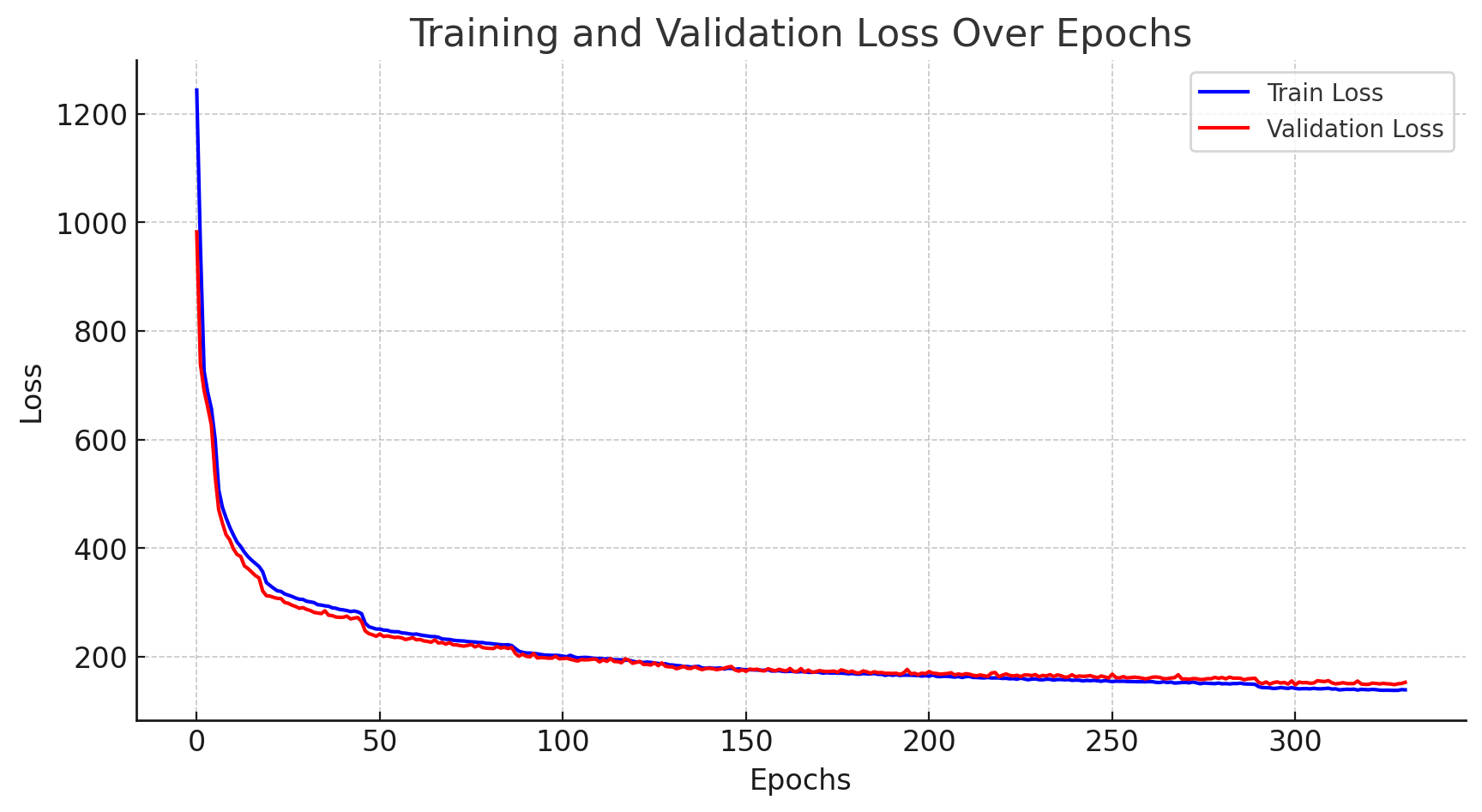
In the final stage, the model, initialized with weights derived from the contrastive learning phase, was fine-tuned to specialize in the generation of high-quality dual-target molecules. The model was initialized from a contrastive learning checkpoint and trained for up to 150 epochs with a batch size of 64, using early stopping with a patience of 30 epochs based on validation loss. Optimization was performed using the AdamW optimizer with an initial learning rate of $5\times{10}^{-6}$.
Sequential learning rate schedulers are used in conjunction with linear warm-ups and cosine decays. A sigmoid annealing schedule was used in the VAE loss function for the Kullback-Leibler (KL) divergence weight, with a maximum value of 0.3, to balance reconstruction and regularization. A partial freezing strategy was implemented to enable focused adaptation, restricting training to the final transformer block of the SMILES-GPT decoder and specific fully-connected layers.

**Figure S12. Training and validation loss curves across 331 epochs during the model pretraining phase.** The steady decline and eventual stabilization of both loss curves indicate effective convergence and generalization, supporting the model’s ability to learn chemically valid representations from the pretraining corpus.


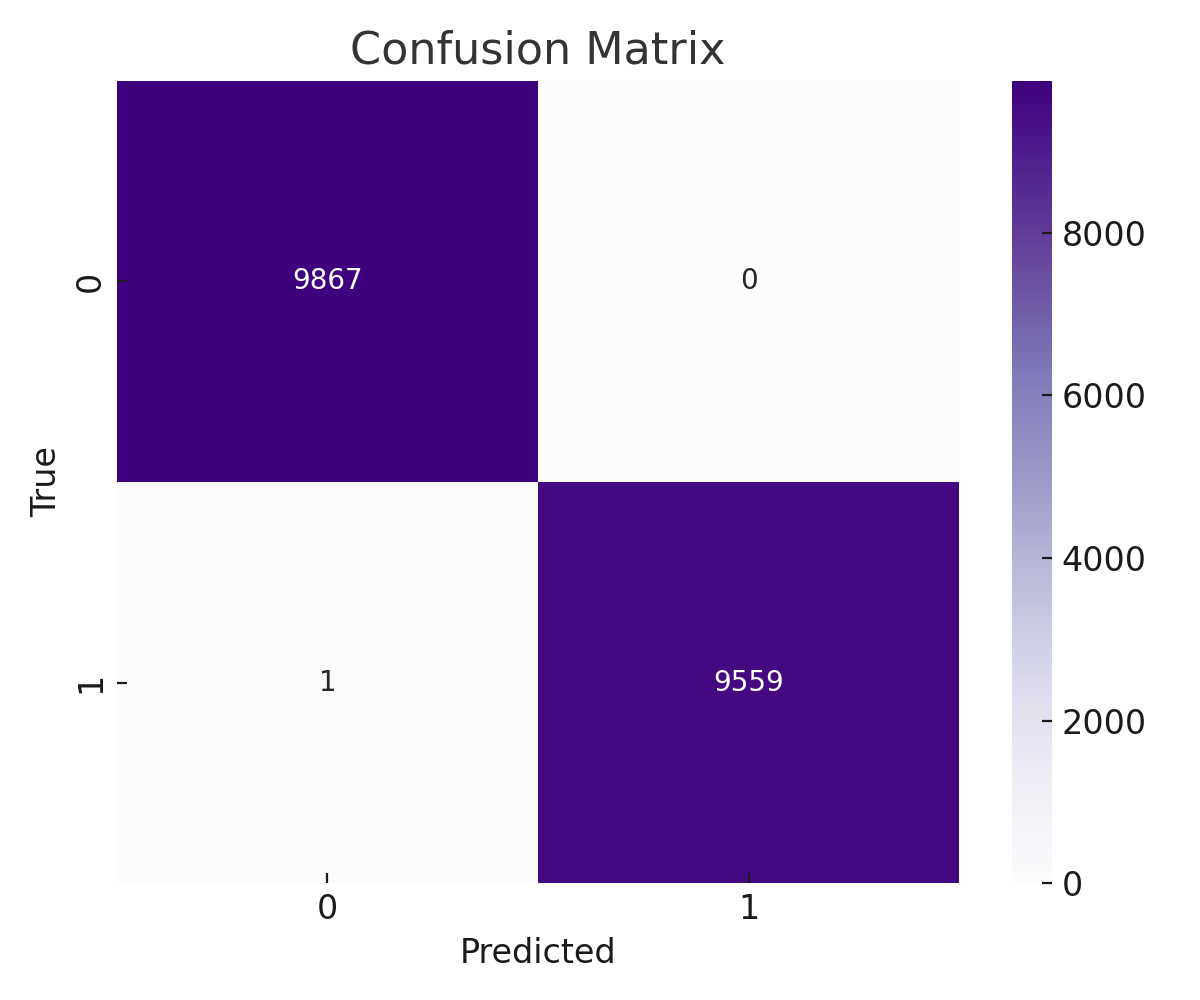

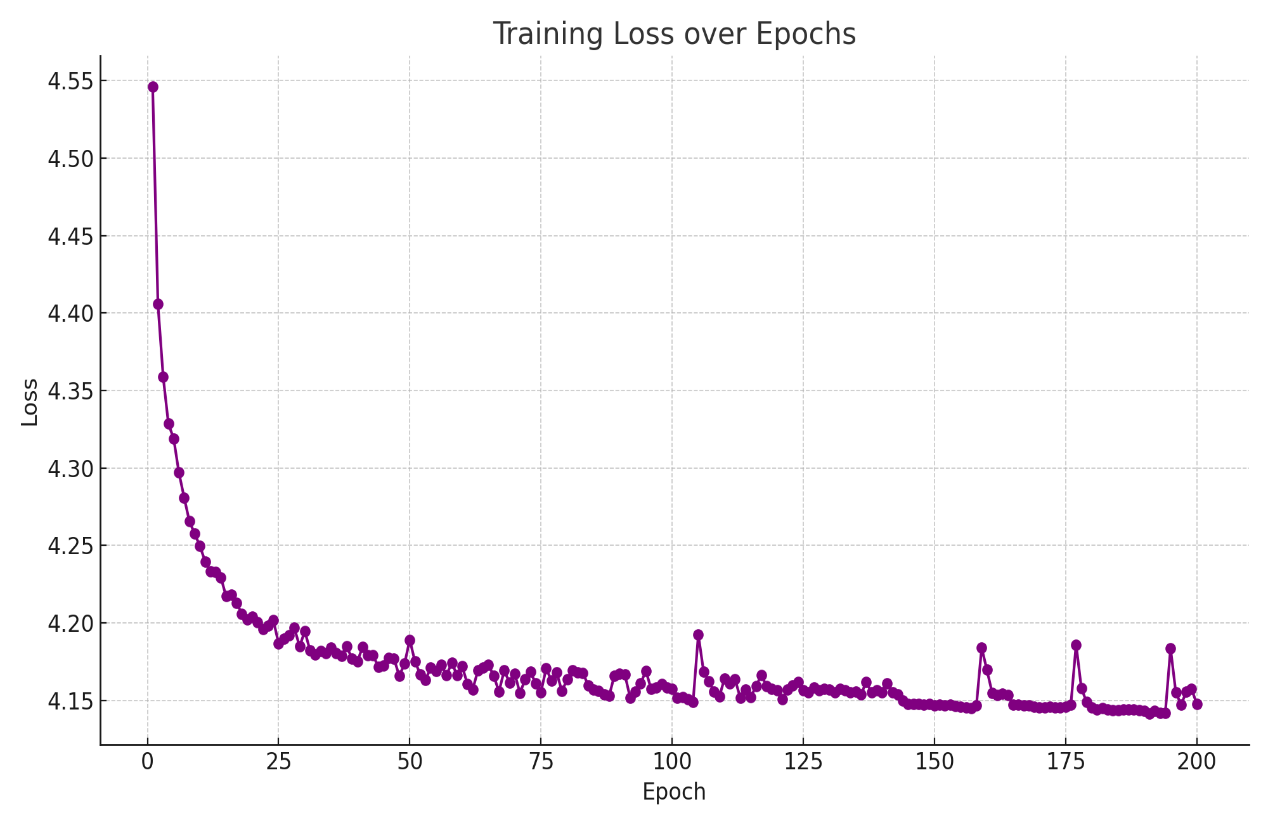


**Figure S13. Training loss trajectory during the supervised contrastive learning phase**

**Figure S14. Confusion matrix summarizing the classification performance of the logistic regression model trained on latent embeddings from the contrastive learning stage.** The model achieves near-perfect discrimination between single-target (class 0) and multi-target (class 1) molecules, with only a single misclassification among 19,427 instances, confirming the robustness of the learned latent representations.

#
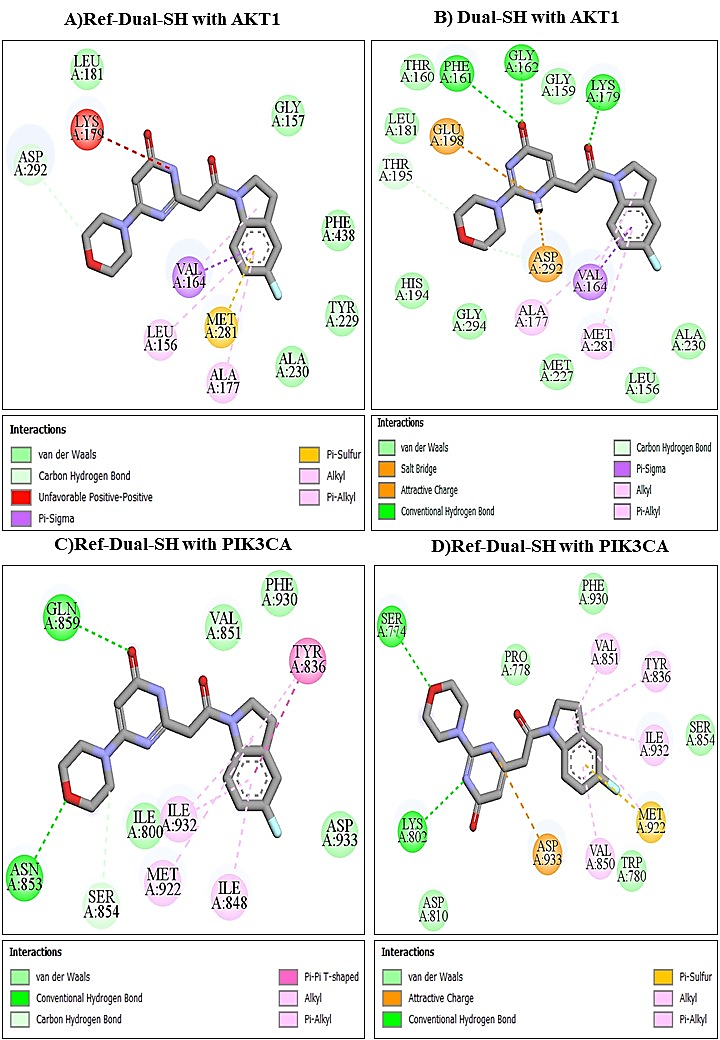
Docking pose and residue-level interaction analysis for scaffold-hopping candidates

**Figure S15-** **Docking interaction profiles for scaffold-hopping dual-target ligands (Dual-SH) and their reference counterparts (Ref-Dual-SH) in AKT1 and PIK3CA.**


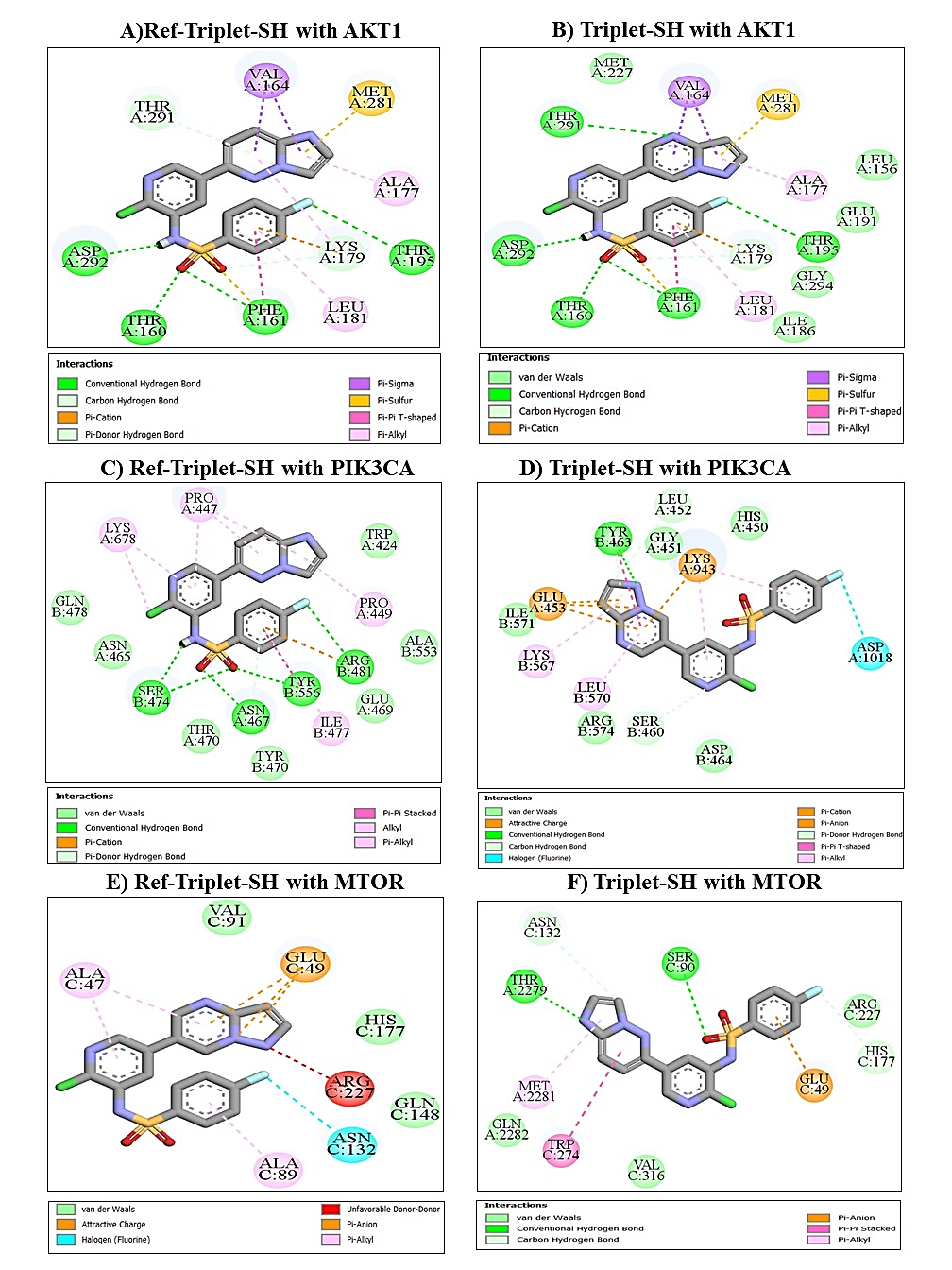


**FigureS16-Mechanistic binding mode comparison of the generated scaffold-hopping candidate Triplet-SH versus the reference inhibitor Ref-Triplet-SH in AKT1, PIK3CA and MTOR.**

# Detailed Generalization Study

## Generalization Study: Cross-Task Robustness in Omics-Driven Phenotypic Drug Design

This study examines the model's ability to generate molecules that induce a desired biological response, as defined by gene expression signatures, rather than being limited to predefined target labels. For this purpose, we employed the base model, Base -BiFormer-GPT, which excludes the supervised contrastive learning stage. Crucially, the training for this task also omits the multi-stage curriculum learning used in the primary experiments, consisting only of pre-training and a subsequent single-step fine-tuning. This approach emphasizes direct generation from a biological signal, rather than clustering a latent space based on multi-target profiles. For the cross-task generalization study, we specifically employed the Base-BiFormer-GPT model, excluding the supervised contrastive and curriculum learning stages. This choice enabled a stringent evaluation of the intrinsic robustness and adaptability of the core architecture. The primary multi-target task and the omics-driven generalization task differ fundamentally in their objectives and data types. The supervised contrastive module structures the latent space based on polypharmacological labels (single- vs. multi-target), which does not apply to gene expression-based profiles in the generalization scenario. Similarly, the curriculum learning strategy designed for dual-to-triplet progression is not suitable for the correlation-based, single-step fine-tuning in the omics task. Evaluating the unmodified base model thus provided a rigorous assessment of the architecture's foundational capabilities. Achieving state-of-the-art results on six out of ten targets in comparison to established benchmarks underscores the versatility and efficacy of the combined BiFormer encoder and SMILES-GPT decoder for diverse drug discovery tasks. Although many generative models prioritize chemical property optimization, the full range of biological responses elicited by a compound is often neglected. To address this, algorithms have been designed to generate novel molecules based on transcriptome profiles. Transcriptome-guided approaches fall into two categories. The first selects source molecules based on correlation with desired gene expression profiles and uses them to train a generative model for producing new active compounds [17-19]. The second category incorporates gene expression data directly as a conditional input to the model [20-26]. This generalization study employs the first approach, shown to produce more realistic molecules by leveraging structures of existing active compounds, ensuring plausible chemical and biological properties. Omics-based techniques offer an advantage by generating hit-like molecules without prior knowledge of ligands or the target protein's 3D structure. Integrating transcriptome data, which reflects cellular responses, directs the model to generate compounds with a higher likelihood of desired therapeutic activity. While GANs and other VAEs have been used for this purpose, our VAE-based framework offers a robust, adaptable alternative. It features a synergistic architecture with a BiFormer encoder, a sparse transformer addressing SMILES semantic discontinuity with high efficiency. Paired with a SMILES-GPT decoder, extensively pre-trained on chemical language, it generates syntactically and semantically valid molecular structures. This integrated method provides a more precise approach to translating complex omics data into novel molecules. This section begins with an overview of the omics-driven experimental design and datasets. Subsequently, we evaluate the reproducibility of generated molecules as ligands and compare our model's performance with state-of-the-art approaches.

### **Datasets and Experimental Methodology for Generalization Analysis**

To establish a comparable data context for evaluating our model against a state-of-the-art (SOTA) approach, the datasets for this generalization study were sourced as outlined in the TRIOMPHE-BOA paper [18], which aggregates data from the ZINC [27], LINCS [28], and ExCAPE-DB databases. While this ensures a standardized data foundation for fair comparison, our omics-based methodology uses a two-phase training protocol: an initial broad pre-training phase to establish molecular syntax, followed by targeted, correlation-driven fine-tuning to generate molecules with specific biological activity.

**Data Preparation for Generalization Task**

The datasets utilized in this study were specifically curated to support the omics-guided generation task:

- **Pre-training Dataset**: The initial pre-training dataset was derived from L1000 and ZINC. To maintain data integrity, 270,099 compounds were refined by removing duplicates and excluding SMILES over 100 characters. After excluding two compounds from the validation set, the final datasets included 243,088 molecules for training and 27,009 for validation.
- **Fine-tuning Datasets:** The fine-tuned sets were identified by correlating two types of transcriptomic profiles from the LINCS database:
- **Gene-Perturbed Profiles**: To characterize the biological consequences of targeting specific proteins, 978-dimensional transcriptome profiles were obtained from the Library of Integrated Network-Based Cellular Signatures (LINCS) database. These profiles, generated using the MCF7 cell line, were acquired through the following methods:
- Gene knockdown for the eight inhibitory targets: AKT1, AKT2, AURKB, CTSK, EGFR, HDAC1, MTOR, and PIK3CA.
- Gene overexpression for the two activation targets: SMAD3 and TP53.

In cases where multiple profiles under different experimental conditions were present, they were averaged to obtain a single target-specific profile.

- **Chemically-Perturbed Profiles:** To establish molecular signatures for correlation, 978-dimensional chemically induced transcriptome profiles were sourced from LINCS. Initially, 16,441 MCF7 cell line profiles at 10 µM dosage were used. This was refined to 16,303 profiles with valid SMILES. A final dataset of 7,677 profiles was curated by excluding compounds with molecular weight ≥ 500, four or more rings, or three or more chiral centers. A reference ligand dataset of known active compounds for ten targets was acquired from ExCAPE-DB.

### **Experimental Methodology for the Generalization Process**

The experimental workflow for this generalization study, shown in Figure S17, followed a two-stage process. First, the Base-BiFormer-GPT model was pre-trained on a large chemical dataset to learn generalizable molecular representations. Then, the model was independently fine-tuned on small, biologically relevant molecular sets identified through transcriptomic correlation.

**Figure S17.Schematic Workflow for the Omics-Driven Generalization Study.**


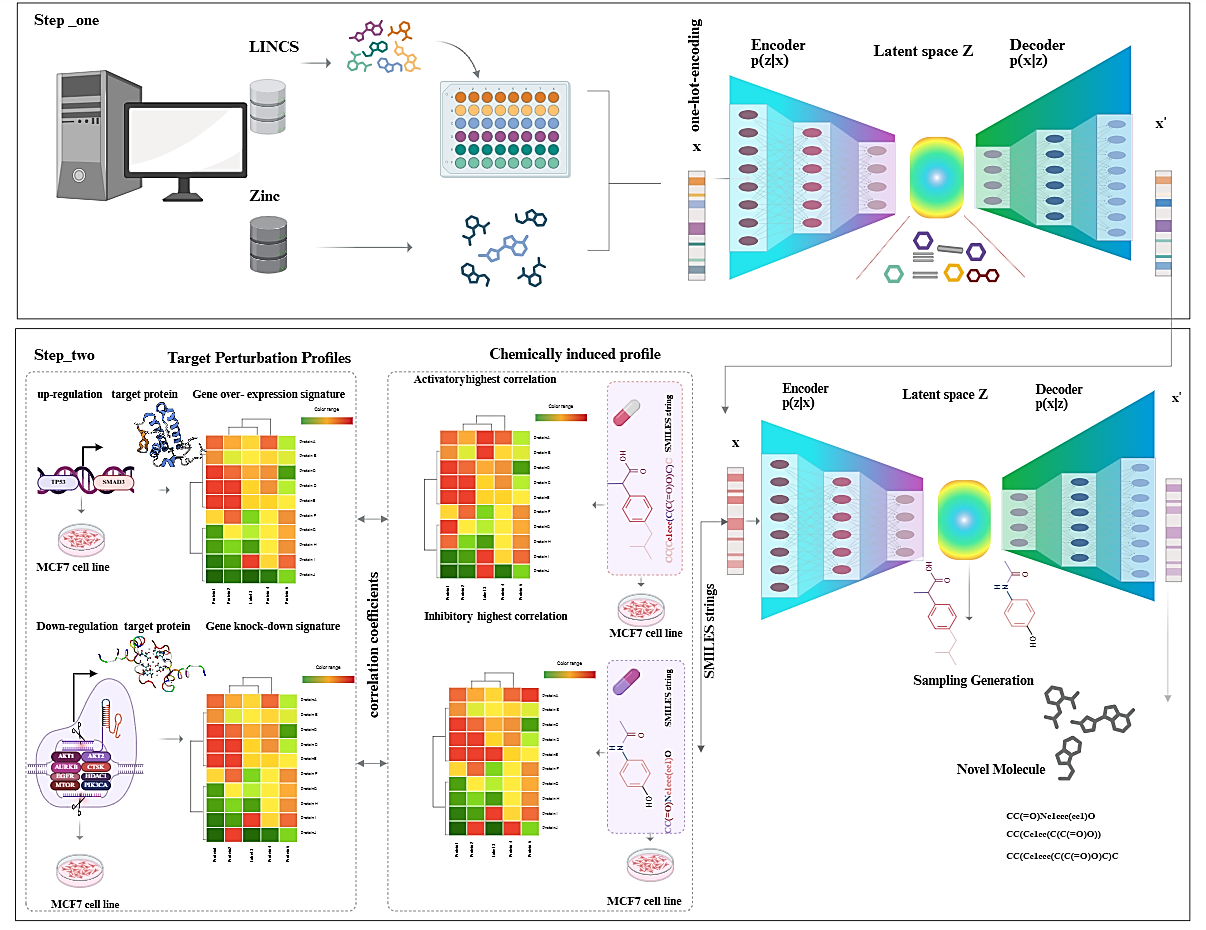


- **Unsupervised Pre-training**

The initial pre-training phase was designed to develop a foundational comprehension of chemical syntax and semantics. This phase utilized a dataset derived from the ZINC and L1000 databases, which was divided into a training set comprising 243,088 molecules and a validation set of 27,009 molecules. In our VAE architecture, the decoder component employs a pre-trained SMILES-GPT model as its generative foundation. To balance the retention of learned chemical knowledge with adaptation to our VAE framework, a hybrid parameter-freezing strategy was employed. Initially, all parameters of the loaded SMILES-GPT model were frozen. Subsequently, the final two Transformer blocks of the GPT decoder were selectively unfrozen, allowing them to be updated during training. Training and validation loss curves for this phase are presented in Supporting Figure S18.The decoder was initialized with weights from the benchmark-5M checkpoint, a model trained on 5 million molecules, chosen over larger variants due to its superior convergence behavior and more stable loss profiles, as observed during preliminary experiments. The optimization employed the ADOPT optimizer, which utilized distinct learning rates for the trainable VAE components and the unfrozen GPT decoder layers, alongside a uniform weight decay. To achieve a balance between reconstruction fidelity and latent space regularization, a KL divergence weight of 0.5 was applied, and a cosine annealing scheduler was used to modulate the learning rate. Although the training was initially set for 1000 epochs, it concluded prematurely at epoch 569 due to an early stopping mechanism (patience=30) that monitored the validation loss.

- **Correlation-Based Fine-Tuning**

The model underwent specialized fine-tuning for each of the ten biological objectives following pre-training. This phase aimed to evaluate the model's capacity to adapt its generative capabilities to a minimal yet highly pertinent data signal. The fine-tuning methodology commenced with a correlation analysis to pinpoint molecules suitable for fine-tuning, conducted separately for each of the ten therapeutic targets, ultimately yielding ten distinct, specialized models. For each of the ten targets, the gene-perturbed profiles (knockdown for inhibitors, overexpression for activators) were correlated with the 7,677 chemically perturbed profiles. A critical feature of this workflow was its application in an extremely low-data regime; for each target, the top three molecules with the highest correlation coefficients were selected to constitute the fine-tuning dataset. Figure S19 illustrates the top three source molecules selected for each therapeutic target, providing representative examples of the molecules used to guide this process. This phase employed more conservative hyperparameters to enable learning from the limited data signal, with a focus on accurately reconstructing the source molecules. Correlation- Based Fine- Tuning Fine-tuning was conducted over a maximum of 20 epochs, utilizing a ReduceLROnPlateau learning rate scheduler with a patience of 10 and an early stopping criterion with a patience of 15. Optimization was performed using lower learning rates of $1\times{10}^{-5}$ for the VAE components and $5\times{10}^{-7}$ for the GPT decoder, with an increased weight decay of $1\times{10}^{-4}.$ A substantially reduced KL divergence weight of 0.1 was used to emphasize the accurate reconstruction of the three source molecules.


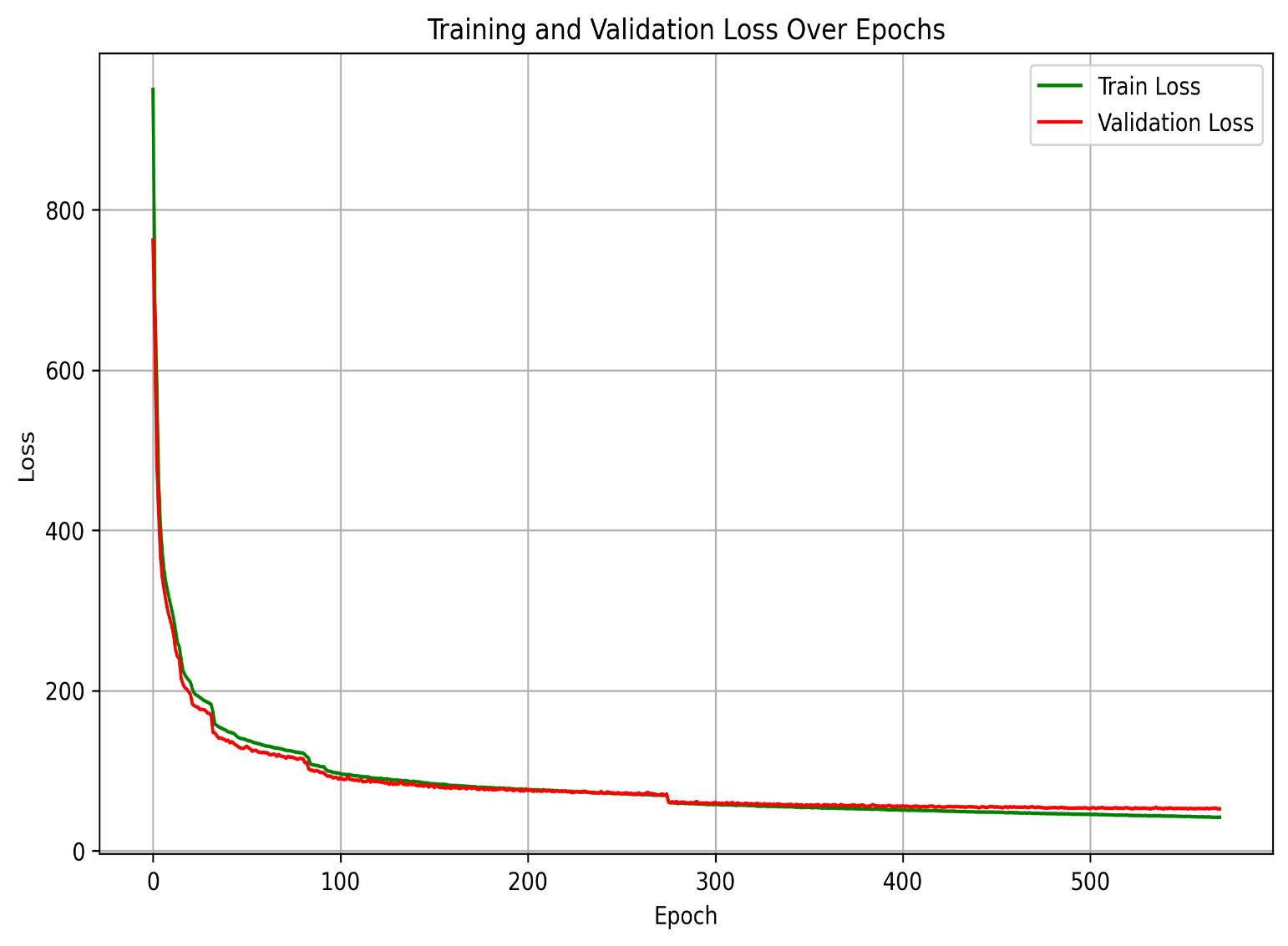


**Figure S18.** **Pre-training Loss Curves for the Generalization Study.**


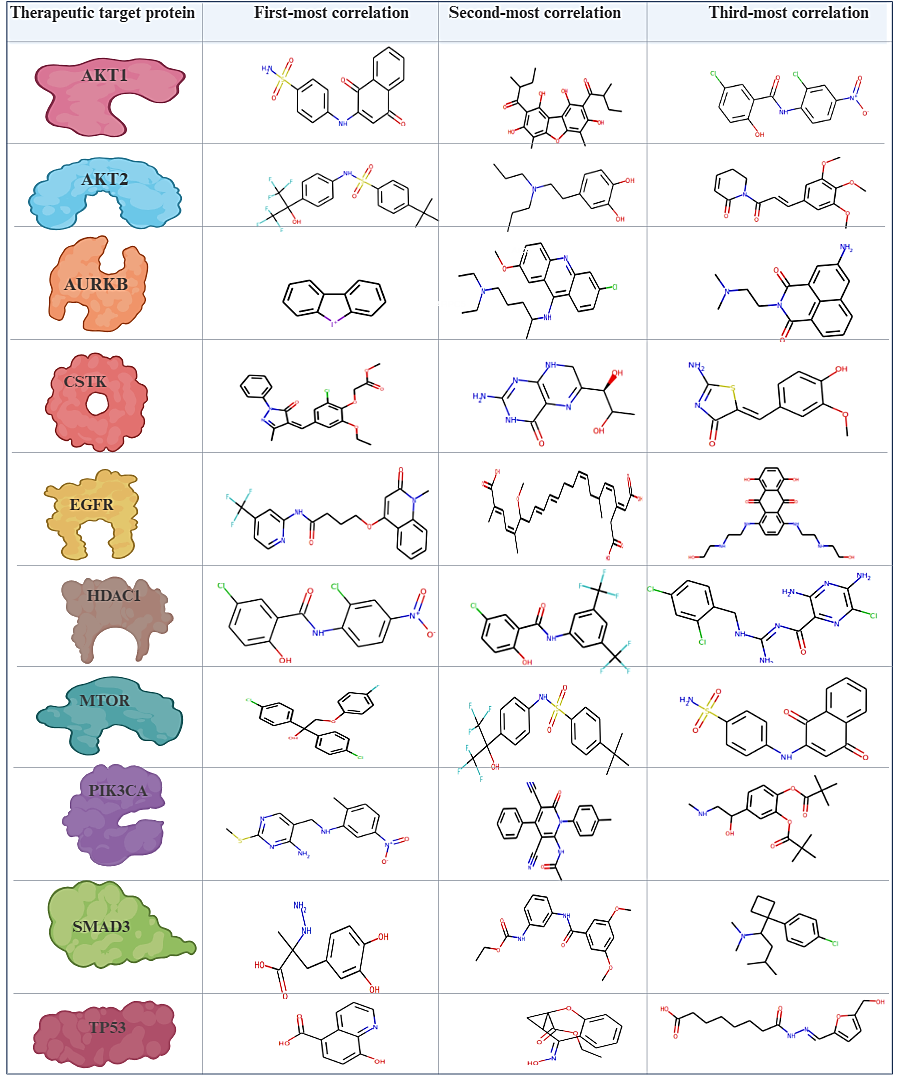
**FigureS19. Molecules Selected for Correlation-Based Fine-Tuning. The chemical structures of the top three source molecules selected for each of the ten therapeutic targets.** These molecules were identified based on having the highest correlation coefficient between their chemically-induced transcriptome profile and the gene perturbation (knockdown for inhibitors, overexpression for activators) profile of the corresponding target. For each independent fine-tuning run, this set of three molecules constituted the entire training dataset used to specialize the pre-trained generative model for a specific biological objective.

### **Assessment of Generated Molecules to Ligand Reproducibility and Generalization Capability**

To evaluate the generalization capability of the MT-BiFormer-GPT model, we computed the maximum structural similarity (Tanimoto coefficient) between its generated molecules and known ligands from ExCAPE-DB across ten targets. Maximum Tanimoto similarity results for each target are summarized in Figure S20. This ligand reproducibility assessment, aligned with TRIOMPHE-BOA’s methodology, provides a standardized benchmark for generation fidelity with biologically validated compounds. By emphasizing similarity between generated molecules and known ligands, we assess the model's ability to generalize beyond training data and capture key pharmacophores for diverse targets. The generated compounds demonstrated varying levels of structural similarity to the known ligands. The highest similarity, 0.5897, was observed for EGFR, while PIK3CA exhibited the lowest at 0.3514. Notably, substantial structural similarities were detected for AKT1 (0.4884), HDAC1 (0.4706), SMAD3 (0.5800), and TP53 (0.5455), all surpassing a similarity score of 0.45. These findings underscore the robustness of the approach in identifying key pharmacophores relevant to these targets.


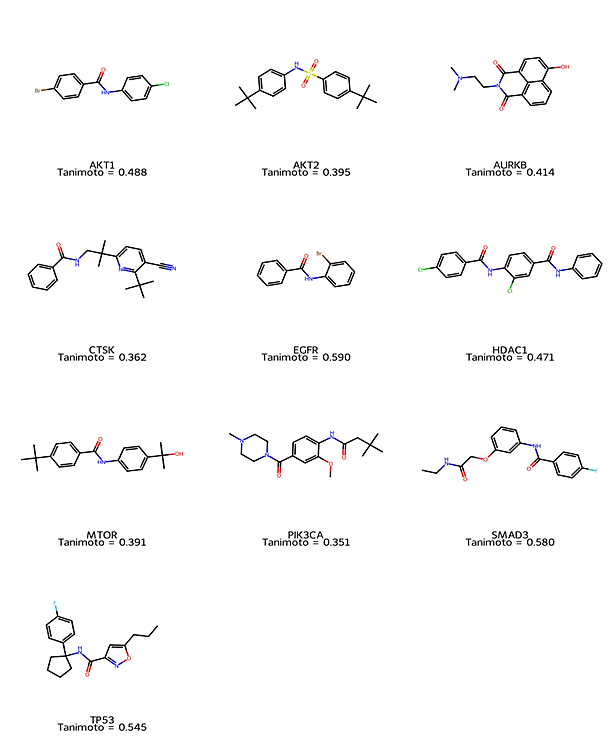


**Figure S20. Generated Molecules with the Highest Tanimoto Similarity to Known Ligands for Each Target Protein.** These compounds are identified as the highest-performing generated molecules for each therapeutic target, exhibiting diverse levels of structural similarity to their respective known ligands.

# REFERENCES

1. Lau, L.Z.X.W.Z.K.W.Z.R., *BiFormer: Vision Transformer with Bi-Level Routing Attention.* 2013.

2. Taniguchi, S., *ADOPT:Modified AdamCanConverge with Any β2 with the Optimal Rate.* 2024.

3. Khosla, P., *Supervised Contrastive Learning.* 2020.

4. Mendez, D., et al., *ChEMBL: towards direct deposition of bioassay data.* Nucleic Acids Res, 2019. **47**(D1): p. D930-D940.

5. Sun, J., et al., *ExCAPE-DB: an integrated large scale dataset facilitating Big Data analysis in chemogenomics.* J Cheminform, 2017. **9**: p. 17.

6. Gilson, M.K., et al., *BindingDB in 2015: A public database for medicinal chemistry, computational chemistry and systems pharmacology.* Nucleic Acids Res, 2016. **44**(D1): p. D1045-53.

7. Kim, S., et al., *PubChem in 2021: new data content and improved web interfaces.* Nucleic acids research, 2021. **49**(D1): p. D1388-D1395.

8. Arus-Pous, J., et al., *Randomized SMILES strings improve the quality of molecular generative models.* J Cheminform, 2019. **11**(1): p. 71.

9. Lu, F., et al., *De novo generation of dual-target ligands using adversarial training and reinforcement learning.* Brief Bioinform, 2021. **22**(6).

10. Ai, C., et al., *MTMol-GPT: De novo multi-target molecular generation with transformer-based generative adversarial imitation learning.* PLoS Comput Biol, 2024. **20**(6): p. e1012229.

11. Polykovskiy, D., et al., *Molecular Sets (MOSES): A Benchmarking Platform for Molecular Generation Models.* Front Pharmacol, 2020. **11**: p. 565644.

12. Landrum, G., *RDKit: Open-source cheminformatics*. 2006, Zenodo.

13. Preuer, K., et al., *Frechet ChemNet Distance: A Metric for Generative Models for Molecules in Drug Discovery.* J Chem Inf Model, 2018. **58**(9): p. 1736-1741.

14. Leo, A., *Partition coefficients and their uses.* Chem Rev 1971;71(6):525–616., 1971.

15. Ertl, P. and A. Schuffenhauer, *Estimation of synthetic accessibility score of drug-like molecules based on molecular complexity and fragment contributions.* J Cheminform, 2009. **1**(1): p. 8.

16. *Hydrophobicity and Central Nervous System Agents:*

*On the Principle of Minimal Hydrophobicity in Drug Design.* journal of pharmaticulchal sceince, 1987.

17. Kaitoh, K. and Y. Yamanishi, *TRIOMPHE: Transcriptome-Based Inference and Generation of Molecules with Desired Phenotypes by Machine Learning.* J Chem Inf Model, 2021. **61**(9): p. 4303-4320.

18. Matsukiyo, Y., C. Yamanaka, and Y. Yamanishi, *De Novo Generation of Chemical Structures of Inhibitor and Activator Candidates for Therapeutic Target Proteins by a Transformer-Based Variational Autoencoder and Bayesian Optimization.* J Chem Inf Model, 2023.

19. Yamanaka, C., et al., *De novo drug design based on patient gene expression profiles via deep learning.* Mol Inform, 2023: p. e2300064.

20. Pereira, T., et al., *Deep generative model for therapeutic targets using transcriptomic disease-associated data-USP7 case study.* Brief Bioinform, 2022. **23**(4).

21. Matsukiyo, Y., A. Tengeiji, C. Li, and Y. Yamanishi, *Transcriptionally Conditional Recurrent Neural Network for De Novo Drug Design.* J Chem Inf Model, 2024. **64**(15): p. 5844-5852.

22. Shayakhmetov, R., et al., *Molecular Generation for Desired Transcriptome Changes With Adversarial Autoencoders.* Front Pharmacol, 2020. **11**: p. 269.

23. Pravalphruekul, N., M. Piriyajitakonkij, P. Phunchongharn, and S. Piyayotai, *De Novo Design of Molecules with Multiaction Potential from Differential Gene Expression using Variational Autoencoder.* J Chem Inf Model, 2023. **63**(13): p. 3999-4011.

24. Das, D., B. Chakrabarty, R. Srinivasan, and A. Roy, *Gex2SGen: Designing Drug-like Molecules from Desired Gene Expression Signatures.* J Chem Inf Model, 2023. **63**(7): p. 1882-1893.

25. Born, J., et al., *PaccMann(RL): De novo generation of hit-like anticancer molecules from transcriptomic data via reinforcement learning.* iScience, 2021. **24**(4): p. 102269.

26. Mendez-Lucio, O., et al., *De novo generation of hit-like molecules from gene expression signatures using artificial intelligence.* Nat Commun, 2020. **11**(1): p. 10.

27. Irwin, J.J., et al., *ZINC: a free tool to discover chemistry for biology.* J Chem Inf Model, 2012. **52**(7): p. 1757-68.

28. Duan, Q., et al., *LINCS Canvas Browser: interactive web app to query, browse and interrogate LINCS L1000 gene expression signatures.* Nucleic Acids Res, 2014. **42**(Web Server issue): p. W449-60.
