# Supplementary material for: MT-ConBiFormer-GPT: multi-target molecular generation for low-data drug discovery via a contrastive BiFormer-GPT architecture and curriculum learning with cross-domain generalization
Source: Brief Bioinform. 2026 May 11;27(3):bbag079. doi: 10.1093/bib/bbag079 (PMC13160422; doi:10.1093/bib/bbag079)
Supplement: bbag079_Supplemental_Files [file bbag079_supplemental_files.zip › Supplementary_Table-revised_bbag079.docx]

# **Supplementary Tables**

# Supplementary Table S1

Table S1- Classification metrics for the H2H and General benchmark's contrastive learning stage, demonstrating higher accuracy of H2H in distinguishing single- and dual-target molecular embeddings.

| **Metric** | **H2H Benchmark** | **General Benchmark** |
| --- | --- | --- |
| **Single target (Class 0)** | P*: **0.9935** / R*: **0.9980** / F1*: **0.9957** | P: 0.9876 / R: 0.9970 / F1: 0.9923 |
| **Dual target (Class 1)** | P: **0.9974** / R: **0.9916** / F1: **0.9945** | P: 0.9969 / R: 0.9875 / F1: 0.9922 |
| **Accuracy** | **0.9952** | 0.9922 |
| **Macro Avg** | P: **0.9954** / R: **0.9948** / F1: **0.9951** | P: 0.9923 / R: 0.9922 / F1: 0.9922 |
| **Weighted Avg** | P: **0.9952** / R: **0.9952** / F1: **0.9952** | P: 0.9923 / R: 0.9922 / F1: 0.9922 |

*Abbreviations: P = Precision, R = Recall, F1 = F1-Score

# Supplementary Table S2

**Table S2: Comparative performance of MT-ConBiFormer-GPT against state-of-the-art models on the DRD2/HTR1A dual-target task using MOSES metrics. Results are reported for both General and Head-to-Head (H2H) benchmark conditions.**

| **Metrics** | **CMolRNN** | **RationaleRL** | **DLGN** | **MTMol-GPT** | **MT-ConBiFormer-GPT** | **MT-ConBiFormer-GPT_H2H** |
| --- | --- | --- | --- | --- | --- | --- |
| Valid | 0.9796 | **0.998** | 0.7801 | 0.8453 | 0.8526 | **1.000** |
| Unique@1000 | 0.4990 | **0.999** | 0.991 | 0.993 | 0.995 | 0.998 |
| Unique@10000 | 0.2628 | **0.9998** | 0.9718 | 0.9545 | 0.982 | 0.992 |
| Novel | 0.6388 | **1.000** | 0.9982 | 0.9836 | **1.000** | **1.000** |
| IntDiv | 0.8248 | 0.8275 | 0.8352 | 0.8349 | **0.887** | **0.889** |
| FCD(DRD2) | 7.2599 | 15.802 | 6.5719 | **5.6181** | 7.275 | **5.5283** |
| SNN(DRD2) | **0.4699** | 0.2869 | 0.3791 | 0.4051 | 0.453 | 0. 4665 |
| Frag (DRD2) | 0.8219 | 0.7311 | 0.9528 | **0.9731** | 0.832 | **0.9821** |
| Scaff (DRD2) | 0.2207 | 0.0039 | 0.2498 | **0.4607** | 0.21105 | **0.50887** |
| FCD(HTR1A) | 6.7187 | 14.9645 | 6.5198 | **5.3975** | 6.2750 | 7.9186 |
| SNN(HTR1A) | 0.4741 | 0.2812 | 0.3911 | 0.4140 | **0. 4853** | 0. 4763 |
| Frag (HTR1A) | 0.8777 | 0.7338 | 0.9448 | 0.9545 | **0.9891** | 0.97497 |
| Scaff (HTR1A) | 0.2591 | 0.0053 | 0.2678 | 0.3915 | **0.4165** | 0.3165 |

# Supplementary Table S3

Table S3- Classification metrics distinguishing single- and multi-target (Dual, Triplet) molecules for the PI3K–AKT–mTOR pathway, based on latent embeddings from supervised contrastive learning.

| **Class Label** | **Precision** | **Recall** | **F1-Score** |
| --- | --- | --- | --- |
| 0 (Single_target) | 0.9999 | 1.0000 | 0.9999 |
| 1 (multi-target) | 1.0000 | 0.9999 | 0.9999 |
| Accuracy |  |  | **0.9999** |
| Macro Avg | 0.9999 | 0.9999 | 0.9999 |
| Weighted Avg | 0.9999 | 0.9999 | 0.9999 |
|  |  |  |  |

# Supplementary Table S4

Table 4-Comparative Evaluation Metrics of MT-ConBiFormer-GPT and MT-BiFormer-GPT Across Dual- and Triplet-Target Generation.

| **Target** | **Model** | **Validity** | **Unique@10 k** | **Novelty** | **FCD/Test** | **SNN/Test** | **Frag/Test** | **IntDiv** |
| --- | --- | --- | --- | --- | --- | --- | --- | --- |
| **Dual** | MT-ConBiFormer-GPT | **0.9931** | **0.9964** | 1.00 | **6.9534** | **0. 4609** | **0.8376** | **0.8766** |
| **Dual** | MT-BiFormer-GPT | 0.9911 | 0.9695 | 1.00 | 7.7852 | 0.3620 | 0.7316 | 0.8740 |
| **Triplet** | MT-ConBiFormer-GPT | **0.9889** | **0.9947** | 1.00 | **6. 7136** | **0.2799** | 0.6283 | **0.8769** |
| **Triplet** | MT-BiFormer-GPT | 0.9092 | 0.9644 | 1.00 | 6. 8372 | 0.2600 | 0.6396 | 0.8753 |

**The bolded values indicate the highest performance for each metric.*

# Supplementary Table S5

**Table S5- Predicted binding affinities for representative dual-target ligands used in the docking analysis.** Ref-Dual-HF and Ref-Dual-SH denote the high-fidelity and scaffold-hopping reference inhibitors selected from the dual-target fine-tuning set, while Dual-HF and Dual-SH are the corresponding MT-ConBiFormer-GPT–generated analogues. “All-Reference” reports the mean docking score over all five dual-target reference inhibitors.

| **Candidate ID** | **Target** | **PDB ID** | **Binding Score (kcal/mol)** |
| --- | --- | --- | --- |
|  |  |  |  |
| Ref-Dual-HF | PIK3CA | 4JPS | -9.5 |
|  | AKT1 | 4EKL | -10 |
| **Dual‑ HF** | PIK3CA | 4JPS | **-9.7** |
|  | AKT1 | 4EKL | **-10.1** |
| Ref-Dual-SH | PIK3CA  AKT1 | 4JPS  4EKL | -9.1  -9.1 |
| **Dual-SH** | PIK3CA  AKT1 | 4JPS  4EKL | **-9.3**  **-9.6** |
| All-References (Average 5 molecules) | PIK3CA | 4JPS | -9.3 |
|  | AKT1 | 4EKL | -9.6 |
|  |  |  |  |
|  |  |  |  |

# Supplementary Table S6

Table S6-Predicted binding affinities for representative triplet-target ligands used in the docking analysis. Ref-Triplet-HF and Ref-Triplet-SH denote the high-fidelity and scaffold-hopping reference inhibitors selected from the triplet-target fine-tuning set, while Triplet-HF and Triplet-SH are the corresponding MT-ConBiFormer-GPT–generated analogues. “All-References” reports the mean docking score over all sixteen triplet-target reference inhibitors.

| **Candidate ID** | **Target** | **PDB ID** | **Binding Score (kcal/mol)** |
| --- | --- | --- | --- |
| Ref-Triplet-HF | PIK3CA | 4JPS | -9.3 |
|  | AKT1 | 4EKL | ‑9.3 |
|  | mTOR | 4JSV | ‑10.0 |
| **Triplet-HF** | PIK3CA | 4JPS | **‑9.4** |
|  | AKT1 | 4EKL | **‑9.5** |
|  | mTOR | 4JSV | **‑10.0** |
| Ref-Triplet-SH | PIK3CA | 4JPS | -9.2 |
|  | AKT1 | 4EKL | ‑9.4 |
|  | mTOR | 4JSV | ‑10.0 |
| **Triplet-SH** | PIK3CA | 4JPS | **-9.4** |
|  | AKT1 | 4EKL | **-9.5** |
|  | mTOR | 4JSV | **-10.0** |
| All-References (Average 16 molecules) | PIK3CA | 4JPS | -9.6 |
|  | AKT1 | 4EKL | -9.5 |
|  | mTOR | 4JSV | -10.7 |

# Supplementary Table S7

Table S7-Comparative Evaluation of Maximum Structural Similarities Across Different Models in the Omics-Driven Generalization Study.

| **Target Protein** | **MT-BiFormer-GPT** | **TRIOMPHE-BOA** | **GAN-based Method** | **TRIOMPHE** |
| --- | --- | --- | --- | --- |
| **AKT1** | **0.488** | 0.423 | 0.317 | 0.417 |
| **AKT2** | **0.395** | 0.37 | 0.289 | 0.351 |
| **AURKB** | 0.414 | 0.500 | 0.31 | 0.49 |
| **CTSK** | **0.362** | 0.31 | 0.275 | 0.34 |
| **EGFR** | **0.59** | 0.386 | 0.298 | 0.306 |
| **HDAC1** | 0.471 | 0.483 | 0.339 | 0.304 |
| **MTOR** | 0.391 | 0.372 | 0.392 | 0.686 |
| **PIK3CA** | **0.351** | 0.344 | 0.261 | 0.324 |
| **SMAD3** | **0.58** | 0.422 | 0.439 | 0.476 |
| **TP53** | 0.545 | 0.62 | 0.457 | 0.53 |
